# Regulation of Intestinal Inflammation by Dietary Fats

**DOI:** 10.3389/fimmu.2020.604989

**Published:** 2021-02-02

**Authors:** Abigail R. Basson, Christy Chen, Filip Sagl, Ashley Trotter, Ilya Bederman, Adrian Gomez-Nguyen, Mark S. Sundrud, Sanja Ilic, Fabio Cominelli, Alex Rodriguez-Palacios

**Affiliations:** ^1^ Division of Gastroenterology and Liver Diseases, School of Medicine, Case Western Reserve University School of Medicine, Cleveland, OH, United States; ^2^ Digestive Health Research Institute, School of Medicine, Case Western Reserve University, Cleveland, OH, United States; ^3^ Cleveland Digestive Diseases Research Core, School of Medicine, Case Western Reserve University, Cleveland, OH, United States; ^4^ Digestive Health Institute, University Hospitals Cleveland Medical Center, Cleveland, OH, United States; ^5^ Department of Hospital Medicine, Pritzker School of Medicine, NorthShore University Health System, Chicago, IL, United States; ^6^ Department of Genetics and Genome Sciences, Case Western Reserve University, Cleveland, OH, United States; ^7^ Department of Immunology and Microbiology, The Scripps Research Institute, Jupiter, FL, United States; ^8^ Department of Human Sciences, Human Nutrition, College of Education and Human Ecology, The Ohio State University, Columbus, OH, United States; ^9^ University Hospitals Research and Education Institute, University Hospitals Cleveland Medical Center, Cleveland, OH, United States

**Keywords:** fatty acids, inflammatory bowel disease, inflammation, high-fat diet, rodent model, obesity, Crohn's disease, ulcerative colitis

## Abstract

With the epidemic of human obesity, dietary fats have increasingly become a focal point of biomedical research. Epidemiological studies indicate that high-fat diets (HFDs), especially those rich in long-chain saturated fatty acids (e.g., Western Diet, National Health Examination survey; NHANES ‘What We Eat in America’ report) have multi-organ pro-inflammatory effects. Experimental studies have confirmed some of these disease associations, and have begun to elaborate mechanisms of disease induction. However, many of the observed effects from epidemiological studies appear to be an over-simplification of the mechanistic complexity that depends on dynamic interactions between the host, the particular fatty acid, and the rather personalized genetics and variability of the gut microbiota. Of interest, experimental studies have shown that certain saturated fats (e.g., lauric and myristic fatty acid-rich coconut oil) could exert the opposite effect; that is, desirable anti-inflammatory and protective mechanisms promoting gut health by unanticipated pathways. Owing to the experimental advantages of laboratory animals for the study of mechanisms under well-controlled dietary settings, we focus this review on the current understanding of how dietary fatty acids impact intestinal biology. We center this discussion on studies from mice and rats, with validation in cell culture systems or human studies. We provide a scoping overview of the most studied diseases mechanisms associated with the induction or prevention of Inflammatory Bowel Disease in rodent models relevant to Crohn’s Disease and Ulcerative Colitis after feeding either high-fat diet (HFD) or feed containing specific fatty acid or other target dietary molecule. Finally, we provide a general outlook on areas that have been largely or scarcely studied, and assess the effects of HFDs on acute and chronic forms of intestinal inflammation.

## Introduction

Many regions of the world are currently affected by an epidemic of obesity and chronic inflammatory disease in humans, which has been, in part, attributed to excessive dietary fat intake (1). In the United States, a ‘Western’ diet which is characteristically high in fat, particularly saturated fats, symbolizes the link between increased availability of fast food diets and public health risk for inflammatory diseases (1–3). Industrialized countries have experienced increased incidence and severity of chronic inflammatory diseases, especially inflammatory bowel disease (IBD), which is thought to be triggered by complex and dynamic interactions between diet, lifestyle, host genetics, the immune system and gut microbiota (4). The IBD subtypes Crohn’s disease (CD) and ulcerative colitis (UC) are chronic inflammatory disorders of the gastrointestinal tract for which there is no cure and which, over time, often require surgical resection of affected portions of the bowel. In the United States, 1.6 million Americans are IBD sufferers (5) who believe that diet, chiefly high-fat diet (HFD) triggers symptoms and flare-ups (6, 7).

Although there are overarching hypotheses linking diet and inflammation, the specific mechanisms mediating such deleterious effects (8), and why some individuals experience them while others do not, are not known. Epidemiological studies have quantified the relationship between fat intake and IBD etiology (3). For example, consuming a diet high in animal fat or polyunsaturated fat (PUFA) has been associated with CD (9), while high intake of monounsaturated or polyunsaturated fats increases the risk of UC (6). Further, obesity has been shown to increase the risk of IBD, while IBD severity (specifically CD) has been found to be greater in obese people (1, 2). Understanding the mechanisms of disease processes is important because it enables the development of strategies to promote human health.

The study of molecular mechanisms of disease in humans is limited by the technical and ethical difficulties, making experimental animals critical avenues for examining the physiological effects of numerous oral and parenteral fatty acid (FA)-derived nutrition combinations. Laboratory rodents, namely mice, exhibit close genetic proximity to the human genome (~90% of mouse genes being homologous to human) (10), thus offering a specific advantage, where precise genetic models of disease can be made. In addition, various rodent models have an increased susceptibility to chronic intestinal inflammation, which worsens with HFDs (11, 12), by immunological mechanisms that also resemble human IBD pathogenesis (*e.g.*, cytokines IL-1β and TNFα, monocyte chemoattractant protein-1 (MCP1), and keratinocyte-derived chemokines) (12). Interestingly, recent evidence now suggests that IBD prevention could intriguingly be achieved by specific dietary FAs, for example, omega-3 (13). This review seeks to summarize proposed mechanisms of disease modulation by dietary FAs, with the ultimate objective to compile peer-reviewed evidence on the mechanisms that could trigger divergent pro- and anti-inflammatory responses.

## Methods of Search

This study was based on a scoping review of published evidence conducted by our group to assess the effects of dietary fats on IBDs in laboratory rodents (rats and mice), and the mechanisms associated with the observed clinical effects on the animal gut. Using systematic search of peer-reviewed reports in PubMed, we identified rodent studies which used a wide array of spontaneous and chemically-induced models of IBD. The data on the type of dietary fats and their direct effect on IBD were extracted from 183 relevant articles published since 1970. We performed an open-term search in PubMed to identify secondary citations. Separate investigators took part in the search and the examination of selected final articles. The initial search assessed all full-text available titles, with the advanced search inclusion criteria of “dietary fat” plus one of the following: inflammatory bowel disease, ulcerative colitis, or Crohn’s disease. The extracted data were assessed for quality and categorized based on the mechanisms associated with either prevention or exacerbation of disease in experimental animals. The data were synthesized for each FA and presented to include chemical structure, the basic nomenclature, and an overview of its effect on intestinal inflammation, followed by a section describing mechanistic principles of modulation.

## Chemical Structure of Fatty Acids

Understanding basic chemical features of dietary fat is important, considering that the pro- or anti-inflammatory effects of FAs are largely dependent on the saturation and length of the FA acyl chains. It is worth emphasizing that any dietary fat, be it animal- or plant-derived, reflects a complex combination of FAs and other molecules that vary with plantation cultivars (*e.g.*, palm tree varieties) and is not always addressed in studies (14–16).

Fatty acids are carboxylic acids that act as principal components of fats such as butter and oils. Fatty acids comprise of a large group of structurally diverse compounds which allows wide range of FA responses to temperature and utilization by the body. Fatty acids are comprised of carbon chains that are either saturated (all carbon-hydrogen bonds are single, thus each carbon is “saturated”) or unsaturated (some carbon-hydrogen bonds are double bonds, thus leaving some carbons “unsaturated”, potentially allowing for saturation, or more hydrogens to be added). Of note, FAs have potent signaling and transcriptional regulatory activities, including in immune cells, while microorganisms use fats primarily as structural components in their cell walls to adapt to environmental changes. Short and unsaturated FAs have lower melting points vis-a-vis long and saturated FAs, and microorganisms adjust to the environmental temperature transitions altering FA composition and adjusting the unsaturation degree, hydrocarbon length, phospholipid charge, and headgroup (17).

**Figure 1:**
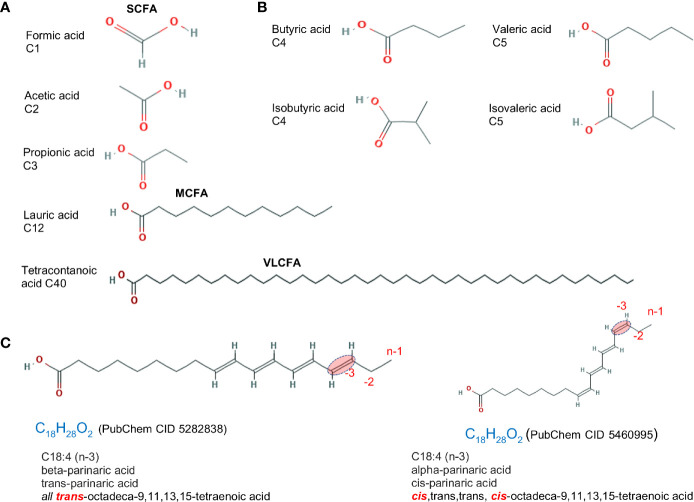
Fatty acid structure for saturated molecules. Examples of differences in fatty acid structure due to carbon length, the presence of methyl branch, and the *cis- trans-* configuration. **(A)** Fatty acids differing based on carbon chain length. **(B)** Fatty acid isomers differing in the addition of methyl branch group. Isoforms rotated to facilitate visualization. **(C)** *cis*- vs. *trans-* structure of a C18:4 n-3 (omega 3). Chemical designation and 2D structures are from PubChem (https://pubchem.ncbi.nlm.nih.gov/).

Traditionally, dietary saturated FAs have been associated with cardiovascular disease; however, the effect of saturation on biology depends on the length of the FA carbon chain and the location and spatial effect of the hydrogen saturation within the carbon chain. An overview of FAs based on saturation/carbon chain length is described below and in **Figure 1A**.

### Fatty Acid Length

Fatty acids are divided into four categories based on chain length: short, medium, long, and very long. Most naturally occurring FAs have 4–28 carbons. *Short chain fatty acids* (SCFA; C:2-C:5) have less than six carbon atoms. These include volatile acetic (C2:0), propionic (C3:0) and butyric (C4:0) FAs, which are mainly produced *via* bacterial fermentation of dietary fiber in the gut and have been extensively studied. SCFAs are beneficial in maintaining intestinal health and considered protective against CD (18). *Medium Chain Fatty Acids* (MCFAs; C:6-C:12) are comprised of 6–12 carbons. Foods like coconut and palm kernel oils are highly enriched with MCFAs (up to 55% of total fat content). As part of medium-chain triglycerides (MCTs), MCFAs are excellent sources of energy, metabolized quickly and can potentially help in weight loss. *Long Chain Fatty Acids* (LCFAs; C:13-C:21) are often referred to as free or non-esterified FAs, i.e. not linked to glycerol backbone. LCFAs are straight chain FAs containing ≥12 carbon atoms, with carbon chains of 16 and 18 constituting the majority of FAs in animal tissues and animal diets. *Very Long Chain Fatty Acids* (VLCFA; ≥C22:0) contain ≥22 carbons and comprise a minority of FAs in a cell (19).

The complexity of the effects of FAs on intestinal inflammation depends on the chemical alterations of the carbon chain, which includes *i)* formation of unsaturated fatty acids by desaturation, i.e. formation of C=C double bonds by the dehydrogenation, which in turn cause *ii) cis* or *trans* configurations, and *iii)* the addition of a methyl group branch (branched FA) (**Figures 1B, C**). A comprehensive list of FAs based on carbon chain length (*e.g.*, C1:0), number of saturated carbons (*e.g.*, C16:4, for four saturated carbons), and the omega designation (*e.g.*, n-3, for omega-3 FA; FA with presence of at least one saturation located three carbons away from the methyl end) is shown in **Table 1**.

**Table 1:** Unsaturated fatty acids and their fatty acid chain length^a^.

	SCFA (≤5 carbons)	MCFA (6–12 carbons)	LCFA (13–21 carbons)	VLCFA (≥22 carbons)
**Saturated** *no double bonds* *(C#:0)*	Formic (C1:0) Acetic (C2:0) Propionic (C3:0) Butyric (C4:0) Valeric (C5:0)	Caproic (C6:0) Enanthic (C7:0) Caprylic (C8:0) Pelargonic (C9:0) Capric (C10:0) Undecylic (C11:0) **Lauric (C12:0)** ^b^	Tridecylic (C13:0) **Myristic (C14:0)** ^b^ Pentadecylic (C15:0) **Palmitic (C16:0)** ^b^^,^^c^ Margaric (C17:0) **Stearic (C18:0)** ^b^ Nonadecylic (C19:0) Arachidic (C20:0) Heneicosylic (C21:0)	Behenic (C22:0) Tricosylic (C23:0) Lignoceric (C24:0) Pentacosylic (C25:0) Cerotic (C26:0) Carboceric (C27:0) Montanic (C28:0) Nonacosylic (C29:0) Melissic (C30:0) Hentriacontylic (C31:0) Lacceroic (C32:0) Psyllic (C33:0) Geddic (C34:0) Ceroplastic (C35:0) Hexatriacontylic (C36:0) Heptatriacontylic (C37:0) Octatriacontylic (C38:0) Nonatriacontylic (C39:0) Tetracontylic (C40:0)
**Unsaturated**^**a**^				
***Omega n-3*** ***(C#:0, n-3)***	–	Octenoic (C8:1) Decenoic (C10:1) Decadienoic(C10:2) Lauroleic (C12:1) Laurolinoleic(C12:2)	Myristovaccenic (C14:1) Myristolinoleic (C14:2) Myristolinolenic (C14:3) Palmitolinolenic (C16:3)^c^ Palmitidonic (C16:4)^c^ **α-Linolenic (C18:3)** ^b^ Stearidonic (C18:4) Dihomo-α-linolenic (C20:3) Eicosatetraenoic (C20:4) **Eicosapentaenoic (C20:5)** ^b^	Clupanodonic (C22:5) **Docosahexaenoic (C22:6)** 9,12,15,18,21-Tetracosapentaenoic (C24:5) 6,9,12,15,18,21-Tetracosahexaenoic (C24:6)
***Omega n-5*** ***(C#:0, n-5)***	–	–	Myristoleic (C14:1) Palmitovaccenic (C16:1)^c^ α-Eleostearic (C18:3) β-Eleostearic (*trans*-C18:3) Punicic (C18:3) 7,10,13-Octadecatrienoic (C18:3) 9,12,15-Eicosatrienoic (C20:3) β-Eicosatetraenoic (C20:4)	–
***Omega n-6*** ***(C#:0, n-6)***	–	–	8-Tetradecenoic (C14:1) 12-Octadecenoic (C18:1) **Linoleic (C18:2)** ^b^ Linolelaidic (*trans*-C18:2) γ-Linolenic (C18:3) Calendic (C18:3) Pinolenic (C18:3) Dihomo-linoleic (C20:2) Dihomo-γ-linolenic (C20:3) **Arachidonic (C20:4)** ^b^	Adrenic (C22:4) Osbond (C22:5)
***Omega n-7*** ***(C#:0 n-7)***	–	**-**	**Palmitoleic (C16:1)** ^b^^,^^c^ Vaccenic (C18:1) Rumenic (C18:2) Paullinic (C20:1) 7,10,13-Eicosatrienoic (C20:3)	**-**
***Omega n-9*** ***(C#:0, n-9)***	–	**-**	**Oleic (C18:1)** ^b^ Elaidic (*trans*-C18:1) Gondoic (C20:1) Erucic (C22:1) Nervonic (C24:1) 8,11-Eicosadienoic (C20:2) Mead (C20:3)	**-**
***Omega n-10***	–	–	Sapienic (C16:1)	–
***Omega n-11***	–	–	Gadoleic (C20:1)	–
***Omega n-12***	–	–	4-Hexadecenoic (C16:1)^c^ Petroselinic (C18:1) 8-Eicosenoic (C20:1)	–

a Note the differences in name designation for each fatty acid once it becomes unsaturated (fatty acids with at least one double bond). Note that there are no short-chain unsaturated FA. Nomenclature varies with the number of unsaturated carbons.

b Most commonly studied dietary fatty acids.

c Example of a fatty acids with the same number of carbons, but with a different configuration and number of carbon saturation. That is, a fatty acid can be either saturated, unsaturated, or classified as omega 3, 5, 6, 7, 9, etc., suggesting that the effect of said fatty acid could also vary based on metabolic alterations.

### Fatty Acid Saturation

Fatty acids that have only single C-C bonds are referred to as saturated, while FAs that contain one or more double bonds (C=C) are referred to as unsaturated. The effects on gut health depend on the degree of fat saturation.

Saturated FAs are derived from animal fats and plant oils, including butter fat, meat fat, and tropical oils (palm, coconut, palm kernel). Common dietary saturated FAs include stearic acid (C18:0; meat, cocoa butter), palmitic acid (C16:0; palm oil, meat), myristic acid (C14:0, cow’s milk, dairy), and lauric acid (C12:0, coconut oil, palm kernel oil, breast milk).

**Figure 2:**
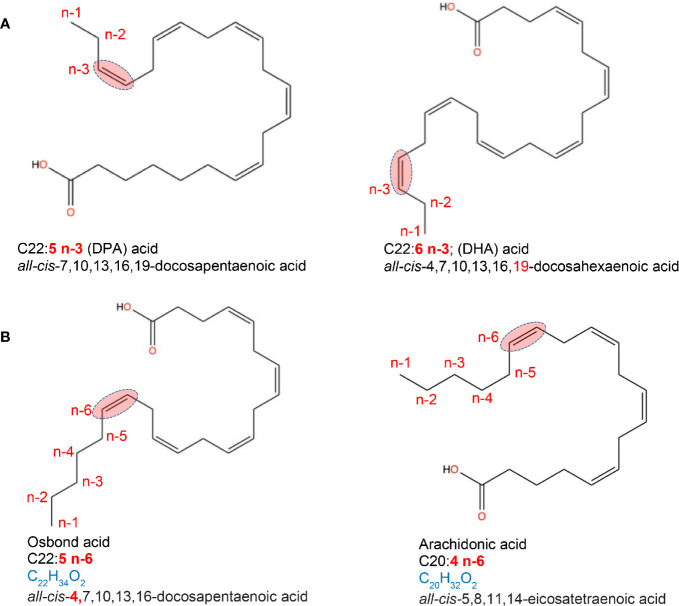
Location of fatty acid saturations. Examples of fatty acids differing in the presence and location of double bond. **(A)** location of saturations for a C22 acid with a double bond in 3rd last carbon (omega-3, n-3). **(B)** location of saturations for a C20 and C22 acid with a double bond in 6th last carbon (omega-6, n-6). Chemical designation and 2D structures are from PubChem (https://pubchem.ncbi.nlm.nih.gov/).

Unsaturated FA can be monounsaturated FA (MUFAs), non-essential FAs that have only one double bond, and polyunsaturated FA (PUFAs), which have two or more double bonds. Common MUFAs include palmitoleic (16:1, n-7), *cis*-vaccenic (18:1, n-7) and oleic acids (18:1, n-9). Oleic acid [C18:1, n-9; ~92% of MUFA consumed in the USA (20)] is the main component of olive oil and macadamia oil. MUFAs are also found in meat/dairy products, although these contain saturated fats. PUFAs are long-chain FAs that include omega-3 (n-3; presence of a double bond in the n-3 position from terminal methyl group) and omega-6 (n-6; presence of a double bond in the n-6 position from the terminal methyl group) FAs. Dietary PUFAs are commonly found in animal and plant-based foods, such as oily fish (salmon), vegetable oils (avocado), and some nuts/seeds. n-3-PUFAs include three FA types; alpha-linoleic acid; ALA (C18:3, n-3; plant oils), eicosapentaenoic (EPA; C20:5, n-3) and docosahexaenoic acid (DHA; C22:6, n-3), both common in marine oils. Of the 11 n-6-PUFAS, linoleic acid (LA; C18:2, n-6) is the shortest-chained and, as with the n-3-PUFA ALA, is an essential FA that cannot be endogenously produced by mammals and thus must be obtained from the diet, namely, plant sources (21, 22) (**Figure 2**).

## Metabolism of Fatty Acids

Structural differences in FA length lead to differences in absorption, transport and tissue destination. SCFAs are water soluble, readily taken up by the cells and mitochondria, and rapidly metabolized by the liver and other peripheral tissues since they are direct precursors for acetyl-CoA (acetic FA), propionyl-CoA (propionate), and butyryl-CoA (butyrate). These CoA derivatives act as direct energy generating molecules in the mitochondria. As the result, SCFAs generated by the bacterial fermentation are present in very low concentrations due to high metabolism. MCFAs are also somewhat water-soluble and do not require transporters to cross the inner mitochondrial membrane and thus are more efficiently absorbed in the gut than LCFA, and more rapidly oxidized in the liver. LCFAs absorption and metabolism are slower since they require special lipoprotein particles (chylomicrons) which are transported through the lymphatic system and allow for greater uptake by the adipose tissue. Biosynthesis of VLCFAs occurs in the endoplasmic reticulum (ER), and unlike MCFAs and LCFAs, VLCFAs are too long to be metabolized in mitochondria. Once inside the cell, MCFAs do not require the carnitine shuttle to move into the mitochondria and appear to preferentially undergo FA oxidation, whereas LCFAs depend on the carnitine shuttle to enter the mitochondria. When long-chain triglycerides are replaced by MCFAs in the diet, differences in metabolic routes appear to promote weight control by stimulating satiety and increased energy expenditure (23).

The metabolism of FAs also depends on saturation. Both linoleic acid (LA, n-6) and alpha-linoleic acid (ALA, n-3) share a common metabolic pathway, wherein ALA competes with LA in delta-6-desaturase binding, which in turn diverts metabolism toward the n-3 PUFAs EPA, DHA and docosapentaenoic acid (DPA; C22:5, n-3) rather than that of pro-inflammatory arachidonic acid (AA; C20:4, n-6) (22). Following this, EPA and AA compete as substrates for lipoxygenase and cyclooxygenase (COX) to generate immunoregulatory eicosanoids including prostaglandins, thromboxanes, prostacyclins, and leukotrienes (LTs) **(** 24). Oleic acid (C18:1, n-9) also plays a role in the metabolism of the essential FAs, serving as a key compound for various metabolic pathways, which may affect disease risk, and has been suggested to compete with LA as a substrate for enzymes involved in the linoleate metabolism (25, 26).

The different activities of AA-derived eicosanoids (pro-inflammatory) compared to those from EPA (anti-inflammatory) are one of the most important mechanisms explaining the anti-inflammatory properties of n-3-PUFAs in inflammatory disorders. This includes the local conversion of AA, LA, EPA and DHA by immune cells (macrophages) to substances known as oxylipins (resolvins, protectins, lipoxins, maresins) (27), potent anti-inflammatory bioactives that reduce tissue inflammation and organ injury (28). Of note, AA intake is associated with IBD development risk (29) and has been shown to accumulate in the IBD colonic mucosa (30), albeit the impact of AA and PUFA metabolism on the treatment/prevention of mucosal inflammation remains controversial (31).

## Overall Fatty Acid Effect on Inflammation Is Variable

Of interest, MCFAs have been associated with anti-microbial/anti-inflammatory functions, whereas LCFAs have been linked to cardiovascular diseases and obesity (17, 32). While the approach to change diet as an intervention has varied considerably between studies, most have involved the replacement or supplementation of a fatty acid/fat (*vs.* complete removal from diet) to study the effect on IBD outcome. Partial or complete replacement of dietary LCFAs by MCFAs has been shown to decrease incidence of spontaneous colitis (33), as well confer protection against chemically-induced gut inflammation, in part, by attenuating pro-inflammatory cytokines and immune cell oxidative stress (enzyme myeloperoxidase; MPO) **(** 34, 35). However, the method of colitis induction can influence outcome; when MCFAs were combined with dextran sodium sulfate (DSS) to form nano-vesicles which fused with the colonic membrane, this may have initiated an inflammatory response, potentially confounding results (36).

Unsaturated FAs (MUFAs, PUFAs) have been associated with lower cardiovascular disease risk, fat mass, waist circumference, blood pressure, and better lipid profiles (higher high-density lipoproteins and lower triglycerides) (37–39). Saturated FAs are associated with increased low-density lipoproteins and higher cardiovascular disease risk, and studies show that, saturated FAs in combination with lipopolysaccharide (LPS) of gram-negative bacteria in the gut, stimulate innate immunity (40).

Several encouraging human and rodent studies have shown that diets rich in n-3-PUFAs can reduce the severity of inflammation in ileum and colon (41), in part, by reducing oxidative stress/modifying the gut microbiota/inflammatory pathways (42–44). Furthermore, studies suggest that partial replacement of LA (n-6) with long chain n-3-PUFAs (at n-6:n-3 ratio of 10) (45) or with medium-chain triglycerides improves experimental colitis (46). Additionally, the ratio of n-3:n-6 plays an important role in disease outcome, with a ratio of 1:3 n-3:n-6 showing the most benefit (47).

In humans, the protective effect of n-3 FAs has been correlated with the decreased production of pro-inflammatory cytokines, through decreased alkaline phosphatase and bile duct injury. However, clinical trials addressing the benefit of n-3-PUFAs in IBD have yielded mixed results, with benefits differing based on the source of PUFA, suggesting differences in anti-inflammatory activity between marine-derived n-3-PUFAS are superior to that derived from plants (48). Addressing the effectiveness of n-3-PUFAs has largely focused on marine-derived fish oils on the notion that they provide EPA and DHA, whereas plant-derived n-3-PUFAs ALA and stearidonic acid are inefficiently converted to long-chain bioactive forms (49).

In mice, n-3-PUFAs have induced a more paradoxical response. Several studies have shown improved inflammatory scores in n-3-PUFA supplemented rodents (50–53), whereas others have noted worsening of intestinal inflammation severity (52, 54). In one study, attenuation of spontaneous ileitis in SAMP1/Yit mice by n-3 PUFA was due to inhibition of monocyte recruitment in the inflamed tissues (55), while two other studies in C57BL/6 mice showed that n-3-PUFAs exacerbated DSS-colitis due to decrease of adiponectin expression, one of which noting no change with n-6-PUFA or control diets (52, 54). In another study, 2,4,6-trinitrobenzenesulfonic acid (TNBS)-colitis rats given n-3-PUFA orally showed inhibition of pro-inflammatory eicosanoids, prostaglandin E2 (PGE2), and leukotriene, similar to treatment with 5-aminosalicylic acid (Peroxisome proliferator-activated receptor gamma; PPARγ agonist) (53), whereas others have suggested a decreasing effect over time, due to T-cell apoptosis/regrowth (56).

## Discrepancies in Treatment Effect Between Animals to Humans

Discrepancies in treatment effect (benefit or harm) between animals and humans may reflect failure of animal models to adequately mimic clinical disease (57, 58). For instance, acute or chemically-induced rodent models of inflammation (*e.g.*, DSS, TNBS) produce disease states within several days and may not reflect a chronic, relapsing disease state. In this regard, adoptive transfer models may prove better suited to study the chronic inflammatory responses (particularly T-cell mediated inflammation), although the lack of B-cells limits direct translation of results to human clinical disease. By comparison, genetically engineered KO mouse models (*e.g.*, IL-10^-/-^ mice), which allow a detailed investigation into mechanistic pathways of IBD, do not reflect the heterogeneous nature of IBD susceptibility (though patients with specific mutations do develop IBD, they are often quite rare) (59). While congenic mice may thus prove advantageous because inflammation develops spontaneously and predictably (*e.g.*, SAMP1/YitFc mouse model), disease pathogenesis is, by definition, a consequence of several factors, making identification of exact mechanisms (without further genetic manipulation) challenging.

The ability for HF research diets to adequately mimic human fat intake is also important to consider given that HFD studies typically use diets with upwards of 60% fat whereas the typical ‘western’ diet contains ~36-40% fat rendering the fat content of experimental diets excessive. Shifts in the non-fat components of the diet to ‘offset’ the increased fat content (*e.g.*, reducing carbohydrate content), as well as FA profiles which do not reflect that of a human diet also affect the translatability of experimental findings to human clinical disease (60–62).

## Factors That Alter the Effect of Fatty Acids (Pro vs. Anti-Inflammatory)

**Figure 3:**
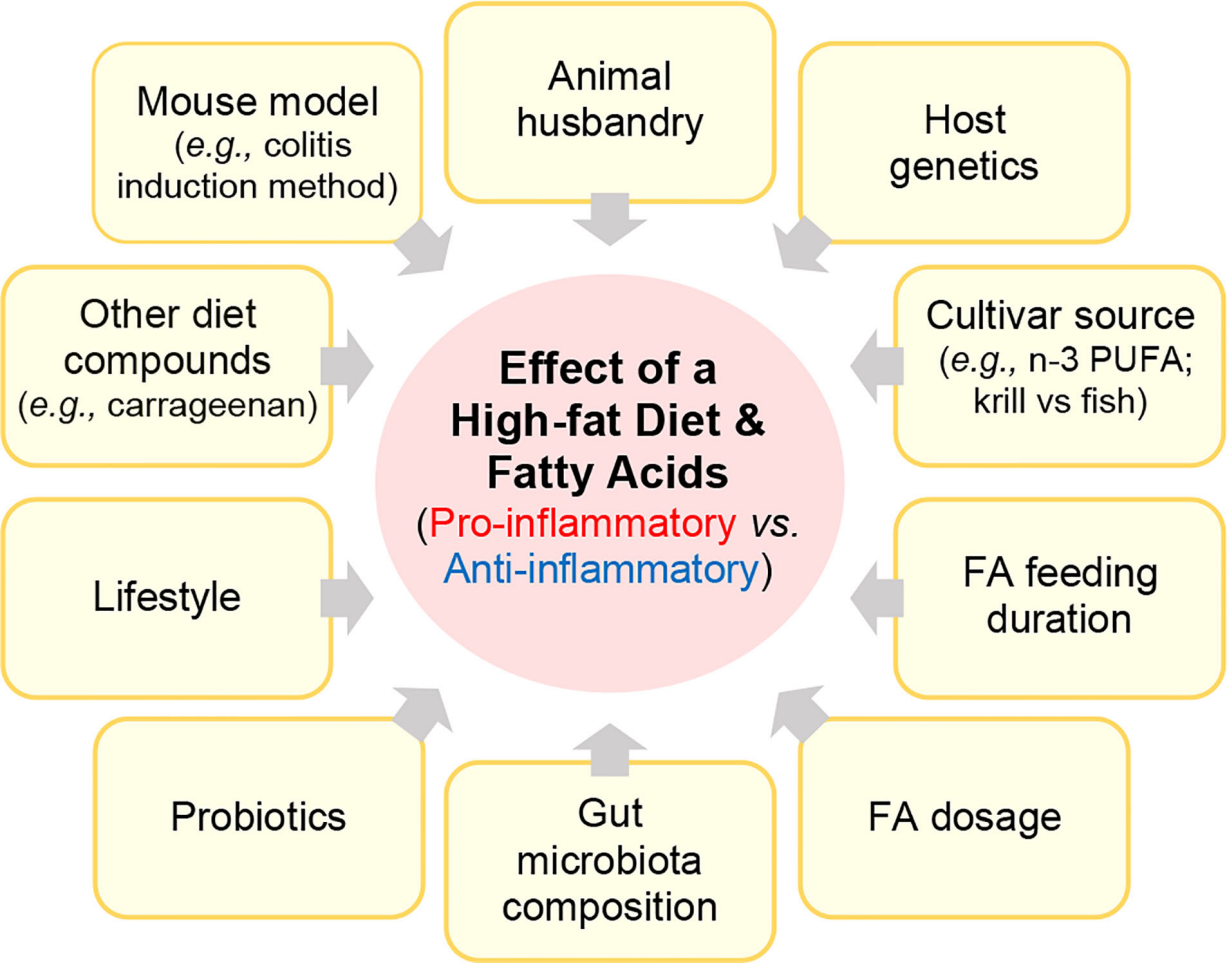
Factors that alter the effect of fatty acids (pro vs. anti-inflammatory).

Numerous rodent studies have investigated how HFD or FAs mediate inflammation in rodent IBD models. However, these studies have varied considerably based on *i)* the IBD mouse model, including the use of spontaneous, or chemically-induced or biologically-induced (*C.rodentium*) injury models, ii) how other factors (diet compounds, lifestyle, drugs, probiotics) could interact with the FA to modulate disease, *iii)* how the feeding trial duration or FA structure/dose affects disease, *iv)* how food sources or processing/manufacturing affect the pro- or anti-inflammatory activity of a FA, *v)* how cultivar or FA source (fish vs. krill) affect outcomes, *vi)* and the role gut microbiota in mediating the effect of a FA (**Figure 3**).

In mice, HFD can induce low-grade inflammation, increasing intestinal permeability (63, 64) and oxidative stress (4, 64–68), which is reversible by anti-inflammatory agents, such as 5-aminosalicylic acid (68), *via* inhibition of NF-κB activation (53). These pro-inflammatory changes in response to HFD often accompany gut microbiota alterations (68–71).

High-fat diets also exacerbate severity of DSS-colitis, independently of obesity (72–75), by disrupting intestinal barrier, upregulating pro-inflammatory cytokines (36, 76, 77) and increasing oxidative stress in colon tissue (78, 79). Exacerbation of mucosal damage by HFDs, particularly saturated fats (*e.g.*, palm oil), is also reported in murine spontaneous ileitis (*Mdr1a* ^-/-^) (80, 81), and colitis (*e.g.*, *Muc2* ^-/-^, *TNF^are^*; defective translational control of TNF mRNA) (72).

The inflammatory potential of HFDs can be enhanced or suppressed by other dietary compounds or lifestyle factors. In one study, the addition of carrageenan, a popular food additive used for decades in the food industry, elicited colitis in unchallenged mice fed a HFD, but not in mice fed standard chow (82). In another study, the combination of a HFD rich in saturated-FAs with antibiotic therapy impaired mitochondrial bioenergetics in the colonic epithelium, triggering gut microbiota alterations to favor pro-inflammatory *Enterobacteriaceae* and in turn, low-grade inflammation (83). By contrast, the addition of exercise (11, 84), probiotics (85–87), or the partial replacement and/or supplementation of ‘anti-inflammatory’ dietary fats (*e.g.*, n-3 for n-6-PUFA), or other nutraceutical/dietary compounds (*e.g.*, red kidney beans, oligosaccharides, choline) (45, 87–89) attenuate the pro-inflammatory effects of a HFD. Studies have explored the role of lifestyle factors such as exercise on intestinal inflammation. In one study, mice with HFD-induced obesity were noted to have less weight gain, improved metabolic disorders, and less expression of inflammatory mediators (preventing colonic inflammation) with increased PPARγ expression in the colon. Given the reversal of this effect by inhibiting PPARγ, the effect seems to be from upregulation of PPARγ (11). In another study, moderate, voluntary exercise was shown to have a beneficial effect in C57BL/6 mice fed a HFD (70% EAF with 200 mg/kg cholesterol) compared to sedentary mice. Exercise significantly decreased TNBS-colitis macroscopic and microscopic severity, increased colonic blood flow, and attenuated plasma TNFα, IL-6, MCP-1, IL-1β and leptin levels in mice fed either a HFD (70% EAF) or a standard regular chow diet compared to their sedentary counterparts (84).

Differences in the amount and type of FA studied, as well as the fat content of basal diets also affect outcome. For instance, one study evaluating diets with varying FA profiles but the same total fat content, found that a 12% saturated fat diet, similar to the standard American diet, lead to the greatest insulin resistance, adiposity, and macrophage infiltration, with 24% and 6% saturated fat diets having significantly lower rates of each of these (90). Further, different FAs can exert the same anti-inflammatory activity *via* different mechanisms. For instance, in *C.Rodentium*-colitis mice, supplementation with either fish or plant oil (flaxseed, ahiflower or sunflower) attenuated colitis, however fish oil reduced lipoxin and leukotriene B4 levels, whereas plant oils increased pro-resolving mediators D, E and T-series resolvins (48). The FA structure is also pivotal in disease outcome. In one study, the glycerol backbone position of palmitate influenced diet effect in *Muc2* deficient mice, with beta-palmitate (palmitic acid bonded to middle position of glycerol backbone) associated with decreased intestinal mucosal damage by inducing an immunosuppressive T-cell response (80). Differences in the absorption/metabolism of substances between animals to humans may limit the ability to translate rodent results to humans, especially relating to doses and duration of studies, with dose-response curves rarely performed in either rodent or human studies.

Highlighting the importance of dietary background for supplement bioactivity, in one *C.rodentium*-colitis study (C57BL/6 mice) flaxseed oil exacerbated colitis in the setting of a reduced-fat diet (~12% energy as fat; EAF), but not a HFD (~36% EAF) (91). Flaxseed (linseed) oil, is a rich source of n-3 ALA (23%), yet also contains lignans, MUFAs (7.5%), PUFAs (6%), saturated-FAs (3.7%) and soluble/insoluble fibers (92). Notably, the exacerbating effects of flaxseed on murine colitis occurred despite increased n-3-PUFAs in intestinal tissues and increased cecal anti-inflammatory SCFAs (91).

With respect to method of IBD induction and basal diet composition/FA content, two partly comparable studies illustrated contrasting effects. Zarepoor et al. **(93) found that ground flaxseed supplementation (10%, 6%, 4%) given to C57BL/6 mice (from Charles River Laboratories, Portage, MI) fed a AIN-93G basal diet worsened DSS-colitis and inflammatory cytokines (colonic tissue and systemically IL-6, IL-1β) and NF-κB related signaling genes (*Nfkb1, Ccl5, Bcl2a1a, Egfr, Relb, Birc3*, and *Atf1*). Whereas Power et al. (** 94) showed, in unchallenged C57BL/6 mice (also from Charles River Laboratories Portage, MI, USA), that flaxseed supplementation (10g/100g AIN-93G basal diet) had beneficial effects on gut barrier integrity (goblet cell density, mucin production, *Muc2* expression and cecal SCFA content, as well as enhanced regenerating islet-derived protein-3-gamma (*RegIIIγ*) and reduced *Muc1* and resistin-like molecule beta (*RELMβ*) mRNA expression indicating that altered microbial defense and injury repair responses are critical (94). While the effect of laboratory rodent providers/facilities on animal microbiota composition have been well documented (95, 96), as a potential explanation, we hypothesize that such discrepant results could have occurred due to the confounding (interacting effect) of the AIN-93G ingredients. That is, in the former study (93), the soybean oil component of the AIN-93G basal diet was replaced with corn oil to “avoid potential anti-inflammatory bioactives” (93), which highlights the important concept of dietary background when designing diets to test supplement bioactivity and the need for studies to ascertain the extent to which various oils, such as soy and corn oil within a basal diet could affect supplement bioactivity.

Supporting the importance of research diet design/manufacturing, and mouse genetics, Cohen et al. (** 97) found no attenuation of intestinal inflammation in IL-10 mice (129 Sv/Ev IL-10^-/-^) fed for 9-weeks (from weaning) a 10% flaxseed oil AIN-93D basal diet (vs. 10% corn oil) (97). However, in BALB/c mice fed a high-fat, high-sugar diet, only low-dose ALA supplementation (150 mg/kg vs. 300 mg/kg) was protective against TNBS-colitis resulting in significantly lower colonic IL-1, IFNγ, IL-4 and IL-2 cytokine production (97).

It is also important to consider all bioactive compounds within a ‘complex’ dietary fat/oil. For instance, sesame oil (7-day supplementation) accelerated healing of colonic inflammation in TNBS-colitis rats by inhibiting inflammation, acid mucin and fibrosis (98). While sesame oil contains ~83-90% MUFAs and PUFAs, mainly linoleic (37%–47%), oleic (35%–43%), palmitic (9%–11%), and stearic acid (5%–10%), the oil (like most oils) contains bioactive phytosterols, tocopherols and a unique class of lignans including sesamin and sesamolin, both shown, when supplemented alone, to exert anti-inflammatory/anti-oxidative activity in experimental IBD (99). Comparably, oleic acid (n-9) sourced from olive oil decreases chronic inflammation by interfering with AA and NF-κB signaling pathways (14), whereas olive oil-derived phenolic compounds protect against oxidative damage in colon cells. Notably, oleic acid and phenolic compounds appear to confer health benefits based on their site of action.

Differences in the source of murine oil (*e.g.*, krill vs. fish oil) or extraction method, including phenolic compounds present between varieties of a single plant-based oil (*e.g.*, olive oil) can exert variable inflammatory responses. For example, in unchallenged C57BL/6 mice, the protective effect of dietary supplementation with different extra-virgin olive oil cultivars on DSS-colitis severity in C57BL/6 mice, including reduction of IL-1β, TGF-β and IL-6 expression levels, was only observed in mice treated with cultivars Ogliarola, Coratina, or Cima di Mola, but not for Peranzana cultivar (14). Such differences have also been noted between krill vs. fish oil, namely differences in structure and antioxidant profiles, which influence intestinal absorption, bioavailability and downstream effects (15, 16). These cultivar studies strikingly illustrate that the effect of a dietary fat source may have altered irreproducible effects on animal experiments and/or susceptibility in humans because cultivars and geographical factors could alter the overall molecular composition of the diet ingredients. However, irrespective of the FA composition, there are well-defined mechanistic patterns of response that are induced by FAs which we summarize below.

## Mechanisms of Action

### Intestinal Mucosa Toxicity and Inflammation

#### Free Fatty Acids, Lipid Droplets, and Cideb

Several studies have reported alteration in free FA levels in intestinal tissues of animals fed a HFD or FA-enriched diet (78, 100, 101). Mammalian cells avoid lipotoxicity from intracellular FAs *via* their esterification and storage as lipid droplets (*i.e.* triglycerides). These lipid droplets are regulated by lipid-droplet associated proteins (LDPs) such as cell death–inducing DFF45-like effector b (*Cideb*) which is abundantly expressed in the intestinal mucosa and helps maintain lipid homeostasis (102, 103). In humans, *Cideb* is a protein-coding gene associated with specific language impairment, that interacts with the DFFA/DFFB complex and activates apoptotic DNA fragmentation. *Cideb* deficiency appears to interfere with lipid metabolism and lipid export from enterocytes leading to excessive lipid accumulation in the mucosa.

In humans, a recent study showed upregulated expression of *Cideb* in the colonic mucosa of patients with UC, as well as in both the protein and messenger RNA Cideb levels of DSS-treated mice (104). Further, administration of a HFD (60% EAF) was found to exacerbate the symptoms of DSS-induced colitis (body weight, histology) observed in *Cideb*-null mice fed a normal diet (10% EAF) compared to their wild-type counterparts (104). Additionally, DSS-treated Cideb-null mice exhibited elevated levels of cytokines IL-1β, IL-6, and TNFα (serum, colon tissues), higher colonic MPO activity and other oxidative stress markers, malondialdehyde, reactive oxygen species (ROS), glutathione (GSH), and superoxide dismutase activity, as well as lipid accumulation in fecal and colon tissues compared to wild-type controls, with more significant increases observed in HFD mice. *In vitro* studies using polarized and Cideb-infected Caco-2 cells treated with oleic acid verified the role of Cideb in lipid metabolism and oxidative stress response of enterocytes. The study revealed reduced lipid accumulation and oxidative stress after the overexpression of *Cideb* in Caco-2 cells, supporting the protective role of *Cideb* against colonic tissue injury, such as in UC (104).

#### Inflammatory Cytokine Profiles Vary With Fatty Acid Content

Studies have shown various effects of cytokine pathways which cannot be easily integrated into a single narrative. However, elevated expression of inflammatory markers such as TNFα, IL-6, IL-1β, and IFNγ (stimulates macrophages to induce innate/adaptive immune responses), among others, and their presence in serum are frequently reported in HFD/FA-enriched rodent studies with/without induction of experimental colitis (14, 50, 51, 75, 79, 105, 106). Obesity-induced inflammation caused by a HFD in mice has also been shown to promote macrophage polarization toward tumor promotion *via* increased IL-6 (107). HFD suppresses IL-10 while inducing TNFα expression *via* regulation of NF-κB in innate and adaptive immune cells (66, 85).

#### Antimicrobial Peptides

The human and animal genome are composed of gene sets that have the ability to produce numerous antimicrobial peptides, some of which have been shown to be modulated by dietary FAs. Cathelicidin antimicrobial peptides are a family of large molecules encoded by single genes, and that are produced in multiple species. Among these, LL-37, FALL-39 (in humans) and mCRAMP (in mice) are found in macrophages, polymorphonuclear leukocytes, neutrophils and epithelial cells, and play a critical role in the innate immune defense against bacterial infection. However, there is also evidence for their role in obesity, and in a model of HFD-induced obesity (45% EAF vs. rodent diet 6% EAF) administration of lentiviral cathelicidin was shown to decrease mesenteric fat and hepatic steatosis by inhibition of the CD36 receptor which in turn suppressed lipid accumulation in adipocytes and hepatic steatosis (108). Lentiviral cathelicidin administration significantly decreased pro-inflammatory cytokine TNFα mRNA expression and sciatica nerve aldose reductase, suggesting that cathelicidin also plays a role in pro-inflammatory gene expression associated with peripheral neuropathy (108).

### Modulation of Pathways

#### Toll-Like Receptor Activation Varies With Diet

Microbial associated pathways can be influenced by diet, which could be recognized by immune cells *via* toll-like receptors (TLR). Thus, TLRs play a key role in innate immunity by recognizing microbe-derived pathogen-associated molecular patterns, which activate immune cell responses. Polymorphisms/mutations in the TLR-receptor/signaling pathways are involved in the etiology and treatment of several inflammatory disorders including IBD (109). Saturated FAs act as ligands of TLR4, and SFA-rich diets have been shown to cause low-grade inflammation and insulin resistance (110, 111). Additionally, HFD-induced changes to the gut microbiota exacerbates inflammation and obesity *via* TLR4 induction and NF-κB (66). Indeed, HFD-fed TLR4-deficient C57BL/10ScNJ mice exhibit attenuated colonic inflammation, reduced pro-inflammatory cytokines (TNFα, IL-1β, IL-6) as well as plasma/fecal endotoxin levels compared to that of C567BL/6 control mice (66). Notably, the dietary phytosterol, β-Sitosterol, which is structurally related to cholesterol and found in plant cell membranes ameliorates HFD-induced colitis in C57BL/6 mice by inhibiting LPS binding to TLR4 in the NF-κB (112).

In one TNBS-colitis model, n-3-PUFA was found to increase *TLR-2* and *IL-1A* gene expression in rat colon tissue, whereas n-9 increased *TLR-4* expression (113). Several studies have shown that by signaling through the G protein-coupled receptor 120 (GPR120), both EPA and DHA exert potent anti-inflammatory effects through inhibition of TNFα receptor and TLR4, inflammatory signaling pathways (114, 115). In adipocytes, EPA has been shown to attenuate palmitate-induced increases in inflammatory gene expression *via* GPR120 by inhibiting the TAK1/TAB1 interaction (114). Notably, TLR regulation by diet also depends on other food ingredients, for instance carrageenan, a red seaweed-derived food additive pervasively used by the food industry as an emulsifier. Increased *TLR-4* expression was noted in C57BL/6 mice fed a 5% carrageenan containing HFD (45% EAF) compared to mice fed a 5% carrageenan low-fat diet (10% EAF) (82). Studies investigating the effect of dietary n-3-PUFA on TLR2 have yielded variable results, with some showing TLR2 downregulation by EPA in mouse adipose stem cells (116) and others reporting no effect from either n-3 or n-9 FA on TLR2 and TLR4 despite their downregulation of IL-6, TNFα and MCP-1 secretion in human adipose tissue and adipocyte cultures (117).

#### Peroxisome Proliferator-Activated Receptor (PPAR) and ABC Transporters

Proliferator-activated receptors (PPARs) are ligand-dependent nuclear receptors for endogenous lipids with 3 isoforms: α, β, and γ, each differing in function and tissue distribution.

**Figure 4:**
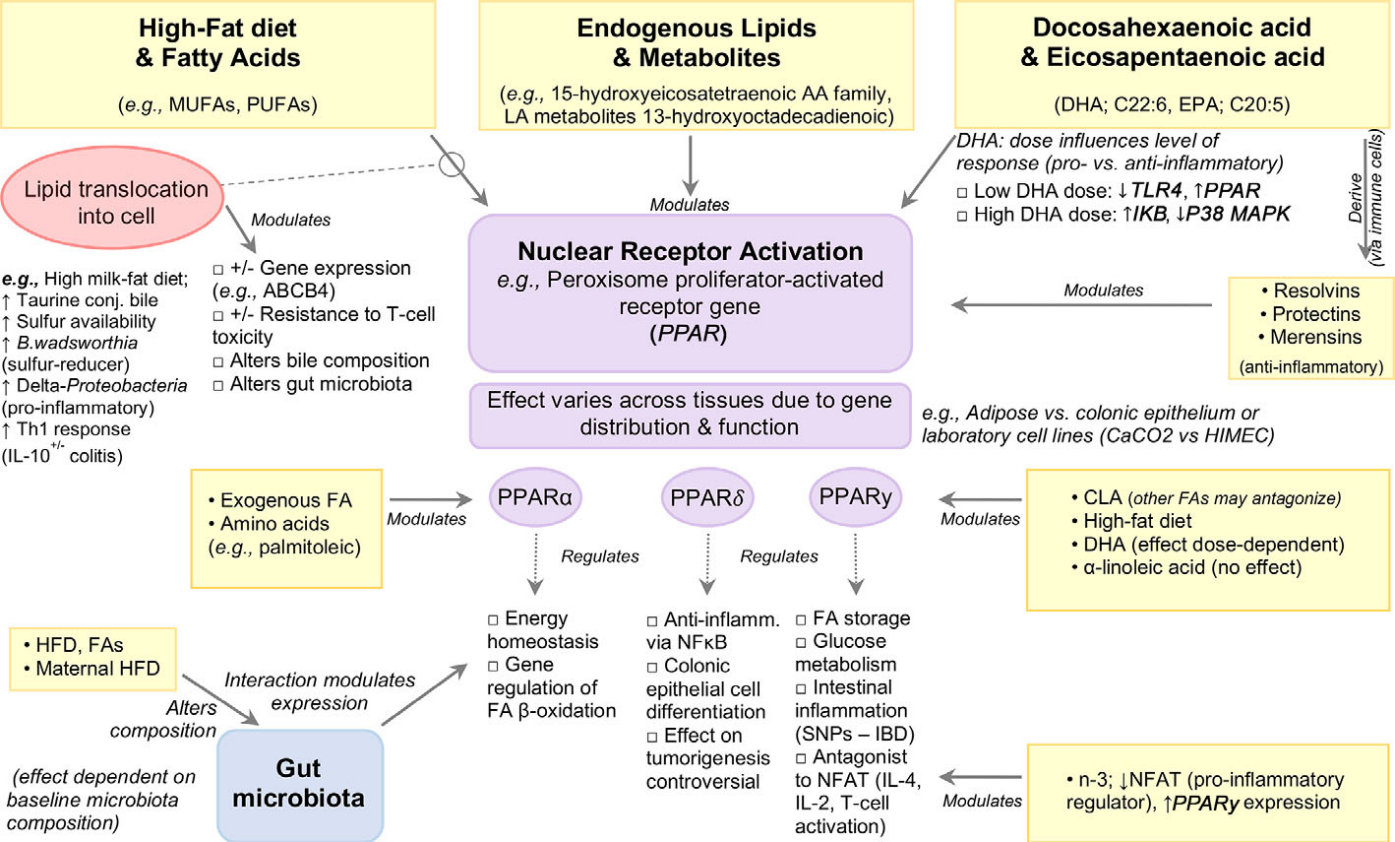
Modulation of Signaling Pathways by High-Fat Diets and Fatty Acids.

PPARγ regulates FA storage and glucose metabolism, and was recently highlighted for its role in intestinal inflammation (118–121), with mutations in the *PPARγ* gene associated with IBD (122, 123). Expressed in adipose tissue and colonic epithelium, PPARγ acts as an antagonist of various transcription factors interfering with their inflammatory pathways, including nuclear factor of activated T-cells (NFAT), an important inducer of pro-inflammatory genes such as *IL-4, IL-2* during T-cell activation (124, 125). In addition, PPARγ activity is modulated by dietary FAs and their metabolites (reviewed elsewhere) (126). The interaction between dietary fats and PPARγ has been well studied for their role in regulating inflammation. Attenuation of TNBS-colitis Sprague-Dawley rats administered dietary n−3-PUFAs (20 mg/day, intragastrically) was associated with enhanced PPARγ expression with a concomitant decrease in *NFAT* expression when compared to trans-FA (13 mg/day) treated rats, indicating that n-3-PUFA inhibits NFAT, potentially *via* PPARγ activation (50). The protective effect of conjugated linoleic acid (CLA) against IBD has been shown *in vitro* and *in vivo* to be mediated through PPARγ activation (22), although other n-PUFAs may antagonize the effects of CLA on PPARγ in experimental colitis (22). By contrast, no effect of dietary ALA-rich oil was seen on PPARγ activation in a TNBS-colitis rat model (127). *In vitro* induction of PPARγ was reported in enterocyte-like Caco-2 cells in response to IL-1β but not in HIMEC cells treated with IL-1β, or LPS-treated human dendritic cells (128, 129). Such discrepancies could be attributed to differences in cell type or DHA dosage, with lower doses serving to inhibit TLR4 signaling and induce PPARγ while higher doses increase *IkB* expression and decrease p38MAPK. Notably, both dosages inhibit intestinal inflammation. It is worth noting that understanding the effects of PUFAs will require better description, owing to the various types of chemical isoforms (*e.g.*, CLA is a family of 28 structural isomers) and the effects of their storage. **Figure 4** illustrates an example of the modulation of signaling pathways by PPAR nuclear receptor activation.

*In vitro* studies have shown that pretreatment of bone marrow-derived dendritic cells with DHA followed by LPS stimulation (TLR4 ligand) profoundly inhibits members of the IL-12 family including IL-12p70, IL-23 and IL-27, an effect mediated by PPARγ and NF-κB inhibition (130, 131). Exposure to DHA also inhibited pro-inflammatory molecule production (IL-6, TNFα, CCL-4) and anti-inflammatory cytokine IL-10 (130), the latter finding in contrast to the upregulatory effects by CLA and subsequent inhibition of LPS-induced IL-12 in murine dendritic cells (132). While the intracellular pathways of DHA activity are not known, *in vitro* evidence suggests PPARγ, which is highly expressed in dendritic cells, macrophages, and T and B-cells, as a possible mediator [reviewed in (133–137)]. Data obtained from *in vitro* and *in vivo* studies also indicate that the anti-inflammatory effect of DHA on endothelial cells is mediated by decreased expression of *VCAM-1* and *VEGFR2* with concomitant reduction in PGE_2_ and LTB_4_ (129). Further, DHA-enriched fish oil has also been shown to enhance B-cell activation *in vivo* which may function to aid pathogen clearance and upregulation of the resolution phase of inflammation, in turn reducing total inflammatory response (138).

PPARα is a major regulator of energy homeostasis and regulation genes involved in beta-oxidation, and is highly expressed in tissues that rapidly oxidize FAs such as liver, heart, kidney, and intestine (139). PPARα is primarily activated *via* ligand binding by endogenous FAs, including AA and palmitoleic acid (140), as well as various other PUFAs and their metabolites, namely members of the 15-hydroxyeicosatetraenoic AA metabolite family and the LA metabolite 13-hydroxyoctadecadienoic (141). In mice, PPARα expression defects have been associated with DSS-induced bile duct injury, which was reversed following DHA supplementation (40 mg/day for 5 days) (142).

PPARδ plays an important role in colonic epithelial cell differentiation (143, 144) and exerts anti-inflammatory activity by inhibiting NF-κB signaling (144). Ligands for PPARδ, including n-3-PUFAs (145, 146), are anti-inflammatory, and at high concentrations PPARδ activation attenuates experimental colitis (147) and intestinal inflammation (148), whereas PPARδ null mice have increased sensitivity to DSS-colitis (149). Relevant evidence highlights the role of PPARδ activation in colon tumorigenesis, however this remains controversial (144, 150–154).

Given that PPARs depend on the entrance/expulsion of FAs from the cell, their effects could be understood/expulsion by factors that alter cell membrane transporters (*e.g.*, ABC transporters). Indeed, Roy et al. (** 155) demonstrated that EPA- and AA-enriched diets downregulated ‘inflammatory’ genes *TNF, IL6, S100A8, FGF7* and *PTGS2*, and upregulated *PPARα, MGLL, MYLK, PPSS23, ABCB4, ABCB1* genes in IL-10^-/-^ mice (B6.129P2-IL10<tm1Cgn>/J) inoculated with intestinal microflora and/or pure cultures of *Enterococcus faecalis* and *E. faecalis* background, compared to C57BL/6J control mice fed AIN-76A diet. Downregulation of the *ABC* genes such as *ABCB1A* and *ABCB1B* has been reported in both human and animal studies suggesting the involvement of ABC transporters during inflammation (156–158). Sundrud et al. (** 159) recently showed ABCB1A modulates the cell activation in the ileum in a bile-dependent manner (*ABCB1* deficiency makes certain T-cell lines prone to bile toxicity) which triggers pro-inflammatory response.

#### PI3K/Akt pathway

Protein kinase B (PKB or Akt) plays a role in cell metabolism, proliferation, growth and survival, and its activation involves phosphoinositide-3-kinase (PI3K) (160). The PI3K/Akt pathway is highly conserved and activation of the pathway is known to inhibit the FOXO3 family. Recent studies have shown that Akt-regulated FOXO phosphorylation increases cellular oxidative stress which in turn induces NF-κB and mTOR activation (161). Additionally, HFD-induced intestinal inflammation was recently shown to be mediated by changes in the Akt-FOXO3 axis (66). Specifically, Akt and FOXO3 phosphorylation increased in mice fed a HFD compared to low-fat diet fed mice, suggesting that NF-κB activation through the Akt-FOXO3 signaling may be associated with intestinal inflammation.

### Fatty Acids and T-Cell Biology

T-cells that infiltrate or reside in the intestinal mucosa sense and respond to pathogen-associated antigens presented by mucosal antigen-presenting cells, most commonly in Peyers patches of the small intestine or in mesenteric lymph nodes, to execute protective inflammatory responses. Mucosal homeostasis requires T-cell tolerance to commensal microbe-derived antigens. A breakdown in T-cell tolerance toward gut commensals is a major determinant of IBD.

#### T-Cell Function

Foxp3+ T regulatory (Treg) cells play critical roles in shaping immune tolerance. Treg cells can develop *via* two major pathways: *i)* as a separate lineage of CD4+ thymocytes (termed natural (n)Treg cells); or *ii)* as mature regulatory cells in the periphery derived from the TGFβ-mediated differentiation from naïve CD4+ T-cells (termed induced (i)Treg cells) (162). iTreg differentiation in the large intestine requires host-microbe interaction with the commensal microbiota, and thus fail to develop in germ-free mice, whereas germ-free animals colonized with defined microbial consortia restores intestinal iTreg development (163–165).

Microbe-derived SCFAs (*e.g.*, butyrate) inhibit histone deacetylase enzymes (HDACs) and epigenetically stabilize *Foxp3* gene expression in developing iTregs (164). Illustratively, butyrate produced by *Faecalibacterium prausnitzii* suppresses experimental colitis *via* HDAC1 inhibition, increasing colonic iTreg cell development/function (166). Microbial bile acid metabolism also modulates gut mucosal iTreg cells. Secondary bile acids, produced through bacterial metabolism of primary bile acids escaping ileal reabsorption (167), promote maintenance of colonic iTregs through the nuclear vitamin D receptor (VDR) (168). Together, bacterial SCFA and bile acid metabolism locally enforces colonic iTreg development to ensure that pro-inflammatory responses to commensal organisms, mediated by IFNу-expressing Th1, IL-4-producing Th2 and/or IL-17A-secreting Th17 effector cells, are tempered by iTreg-mediated immune suppression to prevent onset of chronic T-cell-mediated inflammation.

As an energy source, Tregs prefer FA β-oxidation to generate ATP (169) and it has been speculated that FA oxidation endow iTregs in non-lymphoid tissues, including the gut, a fitness advantage in tissue microenvironments where immune suppression is typically favored. This metabolic preference of iTreg cells for FA oxidation suggests that ingested dietary lipids preferentially regulate intestinal Treg development/function. Indeed, oleic acid has been implicated in promoting Treg function in non-lymphoid tissues, including in visceral adipose tissue and the central nervous system (170); oleic acid is reduced in adipose reservoirs of human multiple sclerosis, a relapsing-remitting autoimmune disorder in which Treg function is impaired, whereas addition of oleic acid to Tregs isolated *ex vivo* from multiple sclerosis patients restores suppressive activity (170). Molecularly, oleic acid enhances FA oxidation and mitochondrial respiration, supporting Foxp3 expression, as well as IL-2/IL-2R/Stat5 signaling, both necessary for Treg maintenance *in vivo (* 170).

#### Glycolysis and FAs

In tumor microenvironments, Tregs are more abundant and have an advantage over T-conventional (Tconv) cells, due to supplemental energy gained *via* lipid metabolism (171). In mouse tumors, Tregs have intracellular lipid accumulation owing to increased FA synthesis, which is enhanced by increased glucose uptake. Therein, both oxidative and glycolytic metabolism contribute to Tregs expansion, which has been corroborated with increased Treg gene signatures on glycolysis and lipid synthesis in humans. Data suggest that signals from the tumor milieu could enable circuitries of glycolysis and FA synthesis/oxidation that confers advantage to Tregs. Less is known on gut wall inflammation, but studies on HFD indicate that certain types of FA result in variable rates of Treg expansion and prevention of IBD, depending on the mouse line (172). Recent studies integrating the gut microbiota with T-cells, have also shown that microbiota-derived SCFAs promote the memory potential of antigen-activated CD8+ T-cells (173), but less is known on how diet-derived LCFA modulate such functions.

T lymphocyte function has been extensively studied using DHA. One study in Smad3^-/-^ colitis-prone mice found that in dietary DHA enhanced LPS-induced B-cell secretion of IL-6 and TNFα, and also increased CD40 expression versus controls. Mice displayed Th2-biasing cytokines as well as cecal IgA, supporting an increased B-cell function (138). In another study, DHA was as effective as sulfasalazine treatment in reducing DSS-induced colitis severity in BALB/c mice, partly by modifying DSS-responsive genes, namely pro-inflammatory cytokines IL-1β, CD14 antigen and TNF receptor superfamily member 1b (*Tnfrsf1b*), membrane remodeling protein (*Mmp-3, -10,* and *-13*) and acute phase protein (*S100a8*) (174). Downregulation of S100a8 was also reported in IL-10 null mice fed an EPA- and AA-enriched diet compared to control mice fed AIN-76A (155).

### Fatty Acid Effect and Modulation Depends on Gut Microbiota

The gut microbiota is shaped by diet and plays an important role in IBD etiology and progression. Most importantly, HFD has been shown to elicit changes in the gut microbiota composition divergent to that of control diets lower in fat, namely increases in alpha diversity and in the *Firmicutes* to *Bacteroidetes* ratio, independent of obesity (66, 79, 175–178). The effect of DSS on gut microbiota composition is also more profound in the setting of a HFD, and has been shown to abrogate the higher abundance of *Firmicutes* to *Bacteroidetes* while increasing the abundance of *Proteobacteria* and *Actinobacteria* (vs. controls) (79). In a DSS-colitis mouse model, a HFD (60% EAF vs. rodent diet, 13% EAF) was associated with 3 phylotypes belonging to *Proteobacteria*; *Trabulsiella*, *Sutterella*, and *Helicobacteraceae*, as well as the phylotype *Atopobioum*, belonging to the phyla *Actinobacteria (* 79). Of these, increased abundance of *Trabulsiella* and *Atopobioum* was also identified in mice fed a HFD without DSS-colitis suggesting that these taxa may exert a colitogenic effect under high-fat feeding conditions. Notably, the administration of colistin (but not vancomycin) ameliorated DSS-colitis severity in HFD mice, indicating that gram-negative bacteria, such as *Proteobacteria* mediate experimental colitis progression in mice fed a HFD (79).

Power et al. (94) demonstrated that flaxseed supplementation (10g/100g AIN-93G vs. AIN-93G) for 3 weeks in unchallenged C57BL/6 mice resulted in a 30-fold reduction in the mucin-degrading bacterium *Akkermansia muciniphila* despite the beneficial effects observed from flaxseed feeding on markers of gut barrier integrity, including mucin production and *Muc2* gene expression. Similar reductions in *A.muciniphila* abundance with increases in *Prevotella* spp. were reported by Gulhane et al. (** 64) following prolonged HFD (46% EAF vs. rodent diet, 11% EAF) in C57BL/6 mice, which was largely reversed following IL-22 treatment (high dose; 100 ng/g) vs. low dose, 20 ng/g recombinant IL-22) in mice. In addition, IL-22 treatment decreased abundance of *Escherichia coli* in a dose-dependent manner, which correlated with decreased serum endotoxin levels. By contrast, Määttänen et al. (91) showed, that the exacerbating effects of ground flaxseed in context of a reduced-fat diet (~12% EAF) fed to *C.rodentium-*colitis C57BL/6 mice decreased relative abundance of *A.muciniphila*, as well as *Parabacteroides distasonis* (irrespective of % energy from fat), a bacterium shown *in vitro* to be dependent on *Akkermansia* presence for its growth, and previously reported to be decreased in inflamed intestinal tissues of IBD patients (179). Although administration of live *P.distasonis* (*via* oral gavage) has been reported to worsen DSS-colitis (180), its cellular components have a protective effect against DSS-colitis (181).

Studies suggest that LPS of gram-negative bacteria stimulate innate immune activity in the presence of saturated FAs (40). Conversely, increased abundance in *Lactobacillus* has been associated with dietary intake of n-3-PUFAs (182), with n-3-PUFA administration to Caco-2 cells shown to promote both *the* growth and adherence of probiotic *Lacticaseibacillus casei* (formerly *Lactobacillus casei*) *(* 183) (Shirota) (184). In this regard, probiotics have been explored as a method to restore intestinal homeostasis in inflammatory states. In a study using *Lactobacillus helveticus* it was noted that the probiotic has varying ability to modulate host physiological function, depending on the diet type, with mice on a western diet showing less *inflammation* than on a standard chow diet (185). One study showed that probiotics corrected inflammation-driven metabolic dysfunction with strong reduction of the colonic expression of inflammatory cytokines TNFα, IL-6, and IFNγ, as well as reserved colonic downregulation of PPARγ, and other ligand-activated nuclear receptors in a TNBS-colitis mouse model (186). Intriguingly, other studies have demonstrated attenuation of HFD-induced (60% EAF; ~90:10% lard: soybean oil) colitis following the administration of lactic acid bacteria (LAB), namely *Latilactobacillus sakei* (formerly *Lactobacillus sakei*) *(* 183) strains (OK67, PK16, S1) (85), as well as in HFD mice treated with IL-10 (anti-inflammatory cytokine) expression-inducing bacteria *Bifidobacterium adolescentis HP1, Limosilactobacillus mucosae HP2* (formerly *Lactobacillus mucosae HP2) (* 183 *)*, and *Weissella cibaria HP3 (* 87). Administration of these bacterial strains appears to attenuate HFD-induced increases in colonic MPO activity, LPS production, NF-κB activation and TNFα expression while enhancing IL-10 expression, in part through inhibition of gut *Proteobacteria* (86, 87).

In addition to the ability of diet to modulate the gut microbiota, several bacterial taxa have demonstrated the ability to generate FAs. Bacterial end-products have exhibited anti-inflammatory effects and have been particularly well characterized in the case of SCFAs. Acetate, propionate, and butyrate acids are synthesized through cleavage of CoA *via* thioesterases, which are ubiquitously found (187, 188). Longer FAs, such as CLA, can be converted from dietary FAs by several genera, particularly, lactobacilli and bifidobacteria *(* 189 *)*. Conversely, reduction of SCFAs has been shown to exert a pro-inflammatory effect. Decreased levels of *Roseburia hominis*, a butyrate producer, is frequently associated with IBD (190). As one might expect, there are also bacteria capable of producing pro-inflammatory FAs. Though bacterial production of non-SCFAs is less studied, bacterial taxa do exist that are capable of synthesizing longer chain FAs. For example, saturated LCFAs from *Prevotella*, lactobacilli, and *Alistipes* increased colitis-mediated death in rats (191). This mechanism of modulation is important to consider especially to try to elucidate the emerging roles of relatively recent gut commensal species such as the *Alistipes* genus which has been shown to have variable effects in humans and animal models (192).

#### Maternal High-Fat Diet

The maternal diet is well known to be one of the major factors influencing offspring microbial composition (193), but more recently, maternal HFD has been shown to modulate susceptibility to diseases, as well as exacerbate offspring susceptibility to chemically induced colitis (194–197) associated with increased IL-1β, IL-6 and IL-17 expression and upregulated NF-κB signaling (194). However, outcomes directly reflect type of FA administered, with one study revealing that the most severe colitis in offspring was from mothers fed (during gestation and lactation) a diet high in safflower oil (~72% 18:2, n-6) compared to those fed diets high in canola oil (18:3, n-3) or high in oleic safflower oil (18:1, n-9) (198).

Maternal HFD has also been shown to result in distinct microbiota differences in offspring compared to that of controls. Xie et al. (195) showed that offspring of C57BL/6 mice fed a HFD (60% EAF) during pregnancy and lactation had distinct differences in bacterial diversity at weaning compared to control offspring (maternal diet of 10% EAF), which continued even after consuming a control diet for 5 weeks after weaning. Furthermore, maternal high fat offspring exhibited significantly inhibited intestinal development and disruption of gut barrier function at 3 weeks of age, as well as accelerated DSS-induced colitis in 8-week-old mice fed a control diet compared to their control counterparts. Inflammation was associated with significant differences in microbiota between offspring groups. Specifically, the maternal HFD offspring had higher abundance of *Echerichia/Shigella, Helicobacter*, and *Oscillibacter*, with decreased abundance of mucosally beneficial *Mucispirillum* and *Barnesiella*, as well as *Anaeroplama* and the SCFA-producing species *Lachnospiraeae inserta sedis* (195). Babu et al. (196) demonstrated that alterations in intestinal microbiota of offspring from breeding mice exposed to a HFD was associated with increased IL-17, as well as increased abundance of *Firmicutes* (primarily *Lactococcus*) with decrease in *Gammaproteobacteria* (primarily *Escherichia*).

Maternal feeding of EPA and DHA (n-3-PUFAs) has also been found beneficial for protecting against inflammation in the intestine of premature pups by regulating eicosanoid and NF-κB related metabolite expression (199). Further, significantly lower incidence of necrotizing enterocolitis (NEC)-like colitis has been reported in pups of n-3-PUFA supplemented mothers (199, 200), associated with reduced IκBα/β levels and elevated PPARγ expression. Although the underlying mechanisms as to how a maternal HFD affects long-term inflammatory outcomes in offspring remains unclear, offspring of mothers exposed to a HFD have been shown to harbor a unique microbiota. In addition, these offspring have increased susceptibility to disrupted mucosal barrier function, low-grade inflammation and experience increased severity of experimentally induced colitis (195, 196). Specifically, one study found expansion of the ILC3 population in the lamina propria of maternal HFD offspring.

### Promotion of Oxidative Stress or Antioxidant Activity

**Figure 5:**
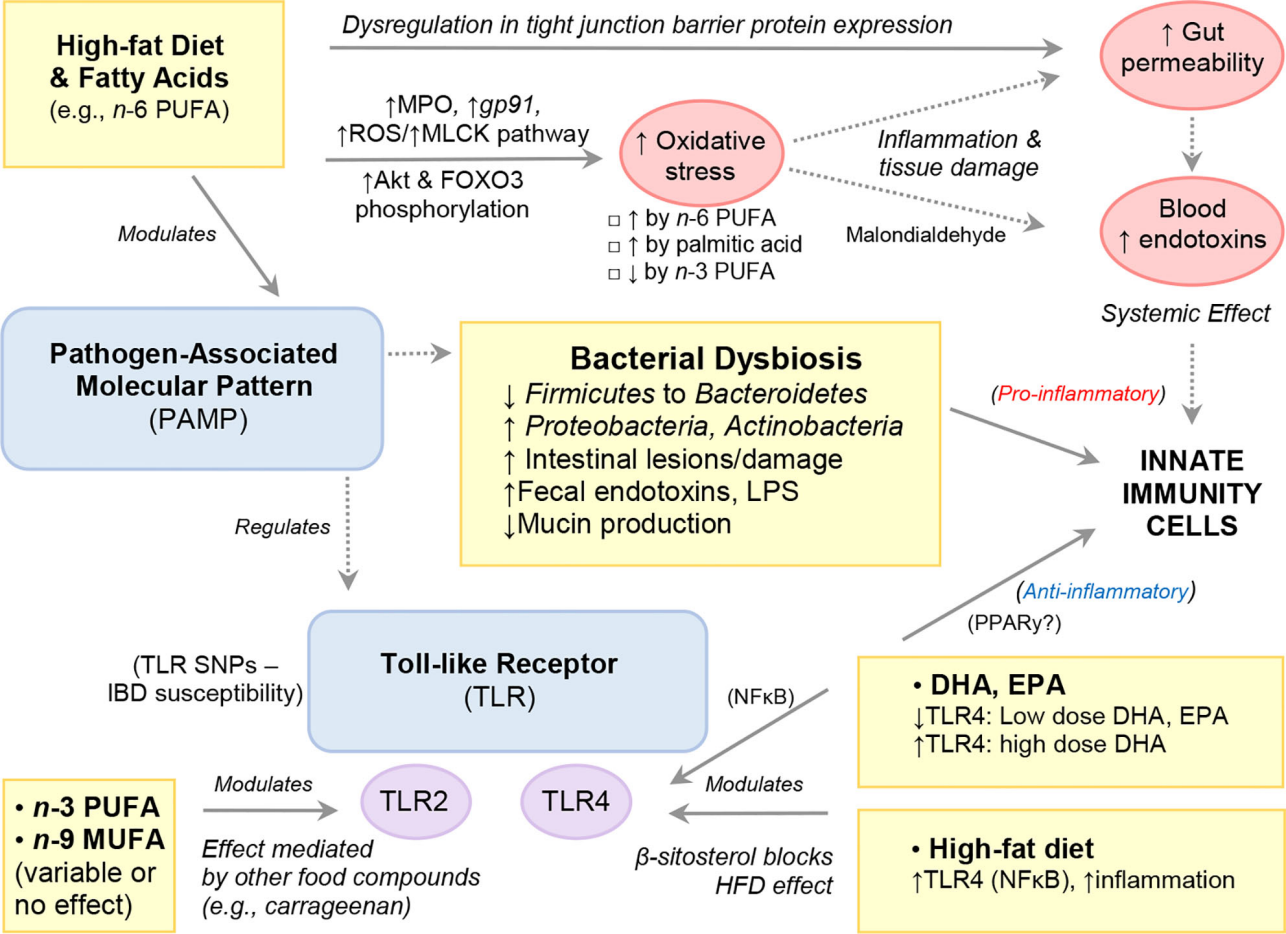
High-Fat Diet and Fatty Acids Modulate Host Immunity via Alterations to Gut Barrier Function and Gut Microbiota Composition.

Numerous diets have long been known to possess an antioxidant effect but in the case of FAs, most of the literature highlights the opposite. That is, the promotion of oxidative stress pathways as a mechanism of induction of inflammation or tissue damage; which is often reported in experimental studies as worsening of histological scores. Oxidative stress is a process by which enzymes and chemical compounds participate in the oxidation and reduction of biological molecules of cell systems. In response to bacterial overload, immune cells have numerous enzymes to trigger oxidation/reduction reactions that have been shown to be modulated by dietary FAs. **Figure 5** illustrates how HFD and FAs can modulate host immunity *via* alterations in gut barrier function and gut microbiota composition.

#### Myeloperoxidase Activity, Glutathione, and iNOS Expression

Neutrophils are phagocytic cells known as first responders in inflammatory reactions that play a key role in host immunity primarily *via* the release of pro-inflammatory enzymes (*e.g.*, MPO), antibacterial molecules (calprotectin, lactoferrin, lipocalin) and DNA NETs to localize infections. Evidence shows that PUFAs, specifically n-6-PUFAs sourced from safflower oil (201), or the n-3-PUFA ALA (127), can elicit changes in neutrophil function and infiltration (decreased), whereas minimal response was seen with fish oil (n-3-PUFA), which had been thought to play a role previously (201). In this context, several rodent studies have shown that HFD, or feeding n-6-PUFA-enriched diet promotes oxidative stress, including increased MPO in the gut (45, 82). In one experimental colitis study, male C57BL/6 mice fed a HFD (60% EAF) for 4 weeks were shown to induce gp91, a NADPH oxidase subunit, and promote production of ROS in both colonic epithelial cells and lamina propria cells compared to their low-fat (10% energy from fat) counterparts following TNBS induction (78). The increased ROS production was accompanied by a concomitant induction of the myosin light chain kinase (MLCK) tight junction pathway as well as increased gut barrier permeability. Increased ROS production and activation of the MLCK pathway was observed *in vivo*, in HCT116 cells cultured with either palmitic acid or a combination of palmitic acid and TNFα. However, this effect was markedly diminished in the presence of a ROS scavenger, suggesting that experimental colitis and mucosal inflammation is promoted by a HFD through aggravation of mucosal oxidative stress, which in turn drives increased gut barrier permeability (78).

Studies have also reported beneficial effects of dietary n-3-PUFAs on oxidative stress. For example, administration of ALA (450 mg/kg) in rats showed a beneficial effect on colonic *iNOS* expression and GSH concentration and inflammatory stress (reduced secretion of TNFα and mRNA level) induced by TNBS-colitis. These protective effects were associated with reduced NF-κB activation as well as reduced lipid mediator concentrations, including leukotriene B_4_ (LTB_4_) and COX2 (127). In another study, dietary olive oil supplemented with n-3-PUFA (fish oil) was found to beneficially decrease colonic *iNOS* expression and GSH concentration in rat colon tissue following DSS-colitis (202).

#### Glutathione Transferase Omega 1 (GST01-1)

Excessive ROS production in the inflammatory response plays a critical role in tissue damage and the progression of inflammatory diseases (203). Studies have recently implicated GSTO1-1 for its TLR4-mediated role in pro-inflammatory response by macrophages (204, 205). TLR4 and MyD88 both play prominent roles in supporting low-grade inflammation in obesity, and deficiency in either protein attenuates obesity and metabolic alterations caused by a HFD (206, 207). Specifically, GSTO1-1 deficient cells failed to upregulate expression of NADPH oxidase 1 and produce ROS following LPS stimulation (208). GSTO1-1 deficient macrophage cells stimulated with LPS were also found unable to produce lactate or dephosphorylate adenosine monophosphate kinase (AMPK; metabolic stress regulator) (205), nor did they accumulate succinate or stabilize HIF1α, two responses important in maintaining pro-inflammatory state of activated macrophages (205, 209). Notably, GSTO1-1 knockout (KO) mice, which are resistant to LPS-induced inflammatory shock (vs. wild type mice), exhibited suppressed pro-inflammatory cytokine expression and attenuated ROS production compared to wild-type mice. Intriguingly, GSTO1-KO mice fed a HFD (23% EAF) for 13 weeks had significantly lower abdominal fat, abdominal adipose tissue inflammation on histology and steatosis compared to their wild type counterparts (210).

#### Glutathione Peroxidase 4

Glutathione peroxidase 4 (GPX4) protects against oxidation of biolipids, referred to as lipid peroxidation, that particularly affects PUFAs with biological membranes. In mice, deletion or inhibition of GPX4 induces ferroptosis, a distinct form of iron-dependent cell death which requires AA (n-6-PUFA) membrane enrichment. In context of the genetic association between GPX4 and CD (211), including evidence of GPX4-restricted AA oxidation in biological membrane (212, 213), a recent study revealed that a PUFA-enriched Western diet triggers GPX4-restricted mucosal inflammation in mice lacking one allele of GPX4 in intestinal epithelial cells (214).

#### Visceral Adipose Tissue Derived Exosomes

Exosomes are endosome-derived nanovesicles that have been recently described as important intracellular communication mediators, especially *via* crosstalk between organs, *via* transfer of encapsulated cargoes such as bioactive lipids, proteins and mRNAs and non-coding RNAs (215–218). Released by healthy cells, exosomes play an important role in the immune system function and have the potential to activate cellular stress and damage (219). Using a DSS-colitis mouse model fed either chow or a HFD it was recently shown that active biogenesis of exosomes occurs in adipose tissue and that these adipose tissue-derived exosomes preferentially circulate to the lamina propria, serving as an important adipokine (220). Further, the HFD-induced obesity altered the miRNA profile of the adipose exosomes, shifting the exosome from having an anti-inflammatory phenotype to that of pro-inflammatory. The intestinal inflammation caused by the circulation of inflammatory exosomes from the obese adipose tissue to the colon was promoted *via* macrophage M1 polarization predominantly *via* the pro-inflammatory cargoes. Most intriguingly, it was shown that colitis could be attenuated by delivering miRNA drugs from the adipose tissue to the lamina propria *via* exosomes encapsulating miR-155 inhibitor, suggesting that targeting the exosomal pathway between obese fat and the intestinal lamina propria could be used to therapeutically manage colitis (220).

#### Endoplasmic Reticulum Stress

Endoplasmic reticulum (ER) stress has been found to influence the pathology of various chronic diseases including IBD (221, 222). Highly secretory cells such as Paneth and goblet cells are extremely prone to ER stress, which activates the unfolded protein response and a cascade of cellular transduction events to restore ER homeostasis (223, 224). Failure of unfolded protein response (UPR) to maintain cellular viability and homeostasis can halt cellular protein synthesis and activate inflammatory signaling and apoptosis. The primary genetic variants within the UPR (*Xbp1, Arg2, Ormdl2*) encoded proteins rely on a robust secretory pathway (*e.g.*, *Muc2, Hlab27*) and mutations in these genes lead to intestinal inflammation (225). In mice, missense mutations in the *MUC2* gene (*e.g.*, Winnie and Eeyore mouse models) result in spontaneous colitis associated with innate and Th17 immune responses, including ER stress which is accentuated by the prolonged HFD feeding in Winnie mice (64). Similarly, prolonged HFD in C57BL/6 mice was shown to induce expression of colonic genes that are markers of ER stress (*sXbp1, Grp78, Edem1*) and oxidative stress (*NOS2*), which corroborated with the increases in ER resident proteins Grp78 and Ire-1B (64).

Specific FAs and cytokines can suppress or exacerbate ER stress in secretory cells (226). For example, IL-10 and IL-22 has been shown to reduce/suppress ER stress *via* their actions on goblet cells (227) and secretory pancreatic B-cells, respectively (226). By comparison, non-esterified FAs such as palmitate administered *in vitro* to human colonic LS174T cells induced significant oxidative and ER stress. This resulted in reduced *Muc2* secretion (mucin production), whereas administration of IL-22 suppressed oxidative and ER stress induced by palmitate (64). Those findings were consistent with *in vivo* studies showing a dose-dependent decrease in ER stress (*sXbp1, Grp78, Edem1*) in response to IL-22 treatment in HFD mice (226). Production of IL-22 is controlled by the aryl hydrocarbon receptor (AhR), an important regulator of metabolism, immune cell homeostasis, and intestinal immunity, activated by dietary ligand binding, namely the phytochemical indole-3-carbinol (228). The AhR regulates IL-22 production *via* intestinal epithelial cells, and AhR signaling has been demonstrated to inhibit inflammation induced by experimental colitis (229), whereas AhR-deficient mice are highly susceptible to DSS-induced colitis (230) suggesting that the AhR plays a key role in resolving intestinal inflammation. Notably, significantly lower AhR activation following feeding of a purified HFD has been reported in mice heterozygous for the AhR repressor gene compared to mice fed a normal, unpurified chow diet (AIN-93G), which contains phytochemicals and flavonoids (231). Furthermore, AhR is targeted by pelargonidins, a type of anthocyanidins thought to be beneficial for overall human health. A synthetic pelargonidin (Mt-P) transactivates AhR, and has been shown, in HFD-fed C57BL/6J mice, to attenuate body weight gain, intestinal and liver inflammation, and ameliorate insulin sensitivity, while worsening liver steatosis, of which were abrogated by gene ablation of AhR (232). Another study in DSS-colitis rats explored the effects of the anthocyanin, pelargonidin 3-glucoside (P3G), on IBD and metabolic syndrome. Findings revealed that P3G treatment attenuated DSS-induced IBD symptoms. Likewise, P3G treatment in rats fed a high-carbohydrate, HFD resulted in attenuation of metabolic syndrome (reduced systolic blood pressure, ventricular stiffness, cardiac and liver structure, abdominal fat, and body weight gain) (233), suggesting that anthocyanidins, specifically pelargonidins, target AhR, decreasing inflammation to attenuate symptoms of IBD and metabolic functions in metabolic syndrome. These findings are relevant considering that diets containing a high content of phytochemicals are generally rich in fruit and vegetables, which are typically lacking in a Western diet.

#### Malondialdehyde

Malondialdehyde is a widely used marker of oxidative lipid injury that results from lipid peroxidation by ROS of PUFAs (234, 235). Malondialdehyde is also a prominent product in Thromboxane A2 synthesis secondary to the metabolism of AA by cyclooxygenase-1 (COX1) or cycloxygenase-2 (COX2) to prostaglandin H2 by various cell types and tissues. Dietary lipid end products from ROS and lipid peroxidases (oxidative stress) such as malondialdehyde are also absorbed into circulation and have been shown to activate inflammatory responses in various tissues, including the gut itself (236). There is also evidence that malondialdehyde is able to regulate insulin through the WNT-pathway, in addition to having mutagenic capability (237).

In TLR4-deficient C57BL/10ScNJ mice, HFD-induced (60% EAF) intestinal inflammation and increased gut permeability was accompanied by the increases in MPO activity and lipid peroxidase levels of malondialdehyde and 4-hydroxy-2-nonenal compared to control C57BL/6 mice fed a low-fat diet (10% EAF) (66). Human studies have yielded contradictory results regarding oxidative stress levels in IBD patients, with some studies reporting significantly higher malondialdehyde levels in plasma of CD patients compared to controls and UC patients, and others showing no difference (238–240).

### Intestinal Permeability

#### Tight Junction Barrier Proteins

Tight junctions are multi-protein junctional complexes which function to seal the paracellular pathway to prevent leakage or translocation of intestinal contents and bacteria across the intestinal epithelium. At least 40 different proteins comprise tight junctions, of which the 3 major transmembrane proteins include occludin, claudins, and junction adhesion molecules (JAM) proteins, which associate with peripheral membrane proteins (*e.g., Z0-1*) located on the intracellular side of the plasma membrane.

Many rodent studies have shown HFD with or without induction of experimental colitis-induced dysregulation in tight junction barrier protein expression (*ZO-1, Claudin, occludin*) in ileal and colonic tissue (66, 79, 241), with concomitant increases in serum endotoxin (consistent with increased gut permeability) (64). Notably, there is evidence that excessive dietary fat and/or the increased luminal bile content, and not genetic obesity, is responsible for the suppression of tight junction proteins and subsequent increased permeability associated with high-fat feeding (242, 243).

However, findings have varied between studies, with some reporting no dietary differences in tight junction expression (113). These discrepancies are possibly due to differences in the amount and type of FAs comprising the diet, duration of diet administration and rodent genetic line. Significant upregulation in expression of *RhoA*, which regulates tight junction assembly and actin organization, has also been reported following feeding HFD (79).

Vitamin D has also been shown to influence gut barrier integrity. Vitamin D is recognized to exert immunomodulatory effects *via* the VDR, and has been shown to exert protective effects in IBD, including amelioration of IBD symptoms in both human and animal following vitamin D supplementation. In one study, vitamin D supplementation (10,000 IU/kg of diet) in C57BL/6N mice fed a HFD (45% energy from fat) attenuated DSS-colitis compared to their counterpart HFD controls supplemented with less vitamin D (1000 IU/kg). Vitamin D supplementation had no effect in the C57BL/6N mice fed a standard diet (10% energy from fat). However, independent of the dietary fat content, all vitamin D-supplemented mice exhibited higher expression of colonic tight junction protein *Cldn1* (P<0.05, but not *Ocln* and *Zo1* mRNA levels P>0.05) whereas expression of colonic *Cyp27b1* (but not VDR) was higher in the HFD vitamin D-supplemented mice vs. their HFD counterparts without supplementation (241).

#### Intraepithelial and Lamina Propria Lymphocytes

In one study, feeding a HFD (56.7% EAF) to C57BL/6 mice for 3 weeks was recently shown to impair the intestinal immune system and increase sensitivity to enteric epithelial damage compared to mice fed a standard diet (13.1% EAF) (244). Specifically, mice fed the HFD exhibited atrophy of the small intestine, colon and gut-associated lymphoid tissue (GALT), with reductions in the number of small intestinal intraepithelial lymphocytes (IEL) and lamina propria lymphocytes (LPL). The latter was also observed in mice within one day of receiving a HFD (244). Effects were independent of changes to the gut microbiota and continued for 2 weeks after returning animals to a standard diet. Intriguingly, reductions in IEL and LPL were also observed in mice supplemented with orally administered FAs, however, this was attenuated upon administration of a lipase inhibitor to reduce luminal free FAs. This suggests that intestinal damage from a HFD was due to the diet-derived free FAs, and that this “intestinal lipotoxicity” may explain, in part, the parallel increase in intestinal diseases, such as IBD, with consumption of a Westernized diet (244).

In another study, aggravated experimental colitis caused by HFD (60% EAF vs. normal fat; 13% EAF) following DSS-colitis in C57BL/6 mice was accompanied by extensive ulceration and inflammation with concomitant crypt regeneration (79). Here, chronic inflammation in high-fat DSS-colitis treated mice was characterized by a lower proportion of TCRγδ T-cells (tissue repair) among IELS while the proportion of TCRαβ T-cells was inversely higher, compared to controls. Both important lymphoid cells among IELs, TCRγδ T-cells and TCRαβ T-cells play a critical role in tissue repair (245) and in controlling intestinal immune responses whose dysregulation is linked to colitis development (246), respectively. High-fat feeding also led to significantly lower proportion of CD8α T-cells which play a unique protective role among IELs (230). Finally, analysis of myeloid cells revealed a higher proportion of CD11b+ monocytes expressing Ly6C in colonic epithelia of HFD fed mice compared to controls suggesting new recruitment of inflammatory monocytes. The concomitant higher proportion of resident CD11b+F4/80+ macrophages in high-fat fed mouse colonic epithelia further suggested a unidirectional change in both myeloid cell subsets (79). There were, however, no significant differences between diet groups in the proportion of pro- or anti-inflammatory cells in the lamina propria.

#### Paneth Cells and Goblet Cells

The intestinal barrier utilizes tightly regulated mechanisms to control and prevent the translocation of intestinal bacteria across the mucosal surface. This includes antimicrobial peptides (AMP) which are produced and secreted by Paneth cells as protective agents against bacterial pathogens, as well as the dense mucus layer of mucins which is produced by goblet cells (*via Muc2*) to serve as a mechanical barrier to prevent bacterial translocation across the epithelial wall (223).

Paneth cell dysfunction (viability and function), reductions in the number and mucin content of goblet cells and subsequent dysbiotic changes in gut microbiota composition has been reported in C57BL/6 mice fed a high-fat (60% EAF vs. control diet of 13% EAF, 25% protein, 62% carbohydrate) (79). Specifically, high-fat feeding resulted in significantly reduced Paneth cell area, reduction of lysozyme content within crypts and decreased expression of procryptdin (AMP exclusively produced by Paneth cells), as well as other AMPs produced at the crypt bottom (*Defcr1, Defcr4, Defa-rs1c*) *(* 79). Mechanistically, reductions in goblet cells was associated with mTORC1 activation, Notch activation and a subsequent downregulation of *Muc2* expression. By comparison, DSS-treated C57BL/6 mice fed a diet rich in extra-virgin olive oil cultivars (Ogliarola, Coratina, Peranzana) exhibited decreased neutrophil infiltration, reduced inflammatory infiltrate and epithelial damage as well as number of dystropohic goblet cells compared to control mice (14). In another study, disruption on mucosal barrier integrity caused by long term high-fat feeding corresponded with significant reduction in *Muc2* mRNA potentially explained by concomitant decrease in the expression of *klf4* and *Spdef*, two transcription factors involved in goblet cell differentiation (64). Similar reductions were observed in *Tff3* mRNA (64), a secreted product of goblet cells that is key to epithelial restoration after injury (247).

### Bile Acids, Prostaglandins, and Resolvins

Bile acids are steroid acids conjugated with taurine or glycine to generate a total of 8 possible conjugated bile acids, which are referred to as bile salts. Bile acids are important to facilitate FA absorption and are synthesized by the liver (primary bile acids) and by bacterial actions in the colon (secondary bile acids).

#### Deoxycholic Acid-Mediates Sphingosine-1-Phosphate Receptor 2

Prolonged exposure to high levels of fecal deoxycholic acid has been shown to disrupt epithelial integrity (248, 249) and contribute to IBD development. In two studies investigating the effect of HFD on bile salts like deoxycholate (known to increase in the colon in individuals on HFDs), wild type mice supplemented with deoxycholate developed inflammation (oxidative, angiogenesis, altered gene expression) (250), whereas *Nos2* KO mice seem to be resistant to these changes (250). More recently, it was shown that excessive fecal deoxycholic acid levels in the gut caused by a HFD contribute to colonic inflammation by dose-dependently upregulating Sphingosine-1-Phosphate Receptor 2 (S1PR2) *via* activation of NLRP3 inflammasome as well as pro-inflammatory cytokine IL-1β production in macrophages (251). Activation of NLRP3 is achieved through downstream stimulation of extracellularly regulated protein kinase signaling pathway (ERK) and subsequent cathepsin B release. In this context, severity of DSS-colitis intestinal inflammation is significantly worsened in mice treated with deoxycholic acid enema but is alleviated by the blockage of S1PR2 as well as inhibition of cathepsin B release, in turn reducing mature IL-1β production.

#### Cyclooxygenase-2

Prostaglandin-endoperoxide synthase 2 (PTGS2) also known as COX2 catalyzes the conversion of AA to pro-inflammatory prostaglandin E2 formation. Cyclooxygenase 2, the inducible form of COX and linked to altered risk of developing IBD (252–254), is the rate limiting step in conversion of AA to prostanoids, pro-inflammatory mediators including protacyclins, prostaglandins and thromboxanes. Both COX2 and COX2-dependent prostaglandin E2 (PGE2) have been associated with maintaining adaptive immune tolerance to dietary antigens (255, 256), with COX2-total KO and COX2-myeloid specific KO mice shown to develop severe CD-like inflammation within the ileo-ceco-colic junctions significantly increasing intestinal permeability when fed a cholate-containing HFD (257, 258). Notably, COX2 can also promote the resolution of inflammation *via* induction of pro-resolving eicosanoid lipoxin A2 (LXA4) (259, 260).

In *fat-1* mice, a transgenic mouse model that can efficiently convert n-6-PUFAs to n-3-PUFAs allowing controlled studies without dietary manipulation, the effect of endogenously synthesized n-3-PUFAs attenuated DSS-induced colonic inflammation accompanied by significant decreases in PGE2 production and COX2 expression as well as decreases in colitis-induced pro-inflammatory cytokines, monocyte chemoattractant proteins (MCP-1, -2, -3) and matrix metalloproteinase 9 (261). Compared to n-6 and n-9 diets, administration of n-3-PUFAs (*e.g.*, ALA, DHA, EPA) has been reported to lower COX2 expression and the production of LTB4 and IL-6 in murine colonic tissue (53, 113, 127), and in endothelial cells *in vitro (* 129).

Diets enriched in EPA and AA have been shown to downregulate PTGS2 gene expression in IL-10^-/-^ mice (262) with concomitant downregulation of IL-6 and TNFα (155), findings consistent with an earlier study showing n-6-PUFA-induced (*e.g.*, AA) inhibition of LPS- PTGS2 protein. Previous studies have shown *in vitro* that EPA, as well as other unsaturated FAs, are potent inhibitors of the AA-induced PTGS2 activity (263). It is possible that AA could also give rise to anti-inflammatory activity given evidence that prostaglandin E2 can suppress macrophage and monocyte production of TNFα and IL-6, as well as inhibit 5-lipoxygenase which in turn disrupting leukotriene X4 production (264).

#### Eicosanoids

Eicosanoids are factors that mediate defensive and inflammatory processes of the gut mucosa and have been shown to increase in experimental colitis. While eicosanoids are known to be regulated by neural and hormonal controls, their local synthesis within the gastrointestinal lumen is influenced by dietary FA intake (5, 265–267). For instance, n-3-PUFA intake has been associated with higher production of EPA eicosanoids (PGE3 and LTC5) and lower AA-derived eicosanoids (6-keto-PGF1 alpha, PGE2, TXB2, LTB4, and LTC4) by the gastric and intestinal mucosa in rats (vs. n-6-PUFA intake) (265), whereas fat-free diets have been shown to reduce eicosanoid production compared to controls (266). The role of eicosanoids in the gastrointestinal tract has been recently reviewed (268)

#### Resolvins, Protectins, and Maresins

Resolvins are anti-inflammatory mediators shown to control and reduce inflammation in a variety of experimental models of inflammatory disorders, mediated, in part, by dendritic cells. Resolvins are derived from n-3-PUFAs, specifically, EPA derives the E-series family of resolvins, while DHA derives the family of D-series resolvins (RvD), protectin D1, and maresins. Both EPA and DHA-derived resolvins participate in anti-inflammatory and pro-inflammatory subsistence *via* signaling pathways including MAPK, NF-κB, PPARу, PI3K, miRNAs, and apoptosis (269). Of the E-series, the three subtypes RvE1, RvE2, and RvE3 have been shown to inhibit leukocyte/neutrophil migration associated with reduced pro-inflammatory cytokine release and increased macrophage phagocytic activity (270). These lipid mediators have been shown to decrease TNFα and IL-6 (271, 272) which may be due to NF-κB signaling *via* its specific G protein-coupled receptor, ChemR23 and leukotriene B4 receptor 1, a receptor of the pro-inflammatory eicosanoid leukotriene B4 (273, 274). Notably, COX inhibitors such as 5-acetylsalicylic acid have been shown to increase formation of AA-derived anti-inflammatory pro-resolution lipoxins, as well as resolvins from n-3-PUFAs such as DHA, supporting the potential for combination therapies using ASA and DHA supplementation (275). The anti-inflammatory role of lipid mediators in the gastrointestinal tract has been recently reviewed (269, 270, 276)

#### Apolipoprotein A-1 (APOA1)

Apolipoprotein A-1 (APOA1) mimetic peptides comprise the main structural protein of high-density lipoprotein. Two of which, 4F and transgenic 6F (Tg6F) have been shown protective of inflammatory diseases, including that 4F, when orally or transgenically administered to low-density lipoprotein receptor-null mice fed a Western diet have the ability to lower pro-inflammatory FA metabolite levels in mouse enterocytes (277). Further, it was recently shown that COX-2 total KO mice fed a cholate-containing HFD and orally administered 4F and Tg6F function to inhibit both LPS and oxidized 1-palmitoyl-2-arachidonoyl-sn-phosphatidylcholine (oxPAPC) signaling in human macrophages and intestinal epithelium, as well as promote the clearance of pro-inflammatory lipids within the gut lumen (278).

### Fatty Acid Effect on Adipokines and Other Hormones

Adipose tissue has been described for its involvement in endocrine (279), metabolic function and more recently for its interaction with the immune system *via* the release of adipokines namely adiponectin, leptin, and ghrelin from fat tissue (280–284). Adipose tissue is also a source of cytokines, including TNFα interleukins (283). Depending on the conditions during their release, these mediators can have pro-inflammatory, anti-inflammatory, or appetite-controlling functions (285, 286). There is also evidence that obesity induces dysregulation of adipokine circulating levels and that this may contribute to obesity-related diseases (287). Further, several studies suggest that adipose tissue-derived mediators, namely increases in circulating TNFα, adiponectin, ghrelin and resistin, with decreases in leptin may affect the pathophysiology of IBD. Discovery of adipokines in fat tissue has led to investigation into their role in inflammatory disorders such as IBD. The role of adipokines in IBD is of particular interest given the involvement of mesenteric ‘creeping’ fat present around inflamed areas of the gut in patients with CD (288, 289), and the recent evidence demonstrating similarities in expression patterns between mesenteric fat adipocytes obtained from obese patients to that of CD patients (290). The role of adipokines in inflammation has been previously reviewed (12, 291).

#### Adiponectin

Adiponectin is a protein hormone released by adipocytes that is involved in glucose regulation and FA oxidation. While several studies have suggested adiponectin to have anti-inflammatory activity (292), more recent studies have implicated its role in the pathophysiology of colitis, although its role remains controversial (293, 294) with some studies reporting an attenuated effect on colitis while others suggest that decreased adiponectin in colon subepithelial myofibroblasts exacerbates colitis (52, 295, 296).

#### Adiponectin Receptor 1 (AdipoR1)

The Adiponectin receptor 1 (AdipoR1) is an important receptor in the fat-intestinal axis during the regulation of inflammation of the colon. Similarities in the expression patterns between mesenteric fat isolated from obese patients and that from patients with IBD have been reported, with inflammation and lipid metabolism pathways showing the greatest overlap (290). Studies looking at the effect of diet-induced obesity on severity of TNBS-colitis and cytokine expression in mouse mesenteric fat suggest that adiponectin receptor 1 aggravates colitis (12). While obesity alone increases pro-inflammatory IL-1β, TNFα, MCP1, and keratinocyte-derived chemokine, obesity decreased the extent to which TNBS-colitis increased IL-2 and IFN-γ in mesenteric adipose and intestinal tissues. *In vitro*, fat-conditioned media lowered AdipoR1 in human colonic epithelial cells (NCM460), while *in vivo* intracolonic silencing of AdipoR1 in mice exacerbated TNBS-induced colitis (12). In another DSS-colitis study, induction of colitis significantly decreased adiponectin and increased expression in both AdipoR1 and adiponectin receptor 2 (AdipoR2) (52). Of interest, findings from McCaskey et al. (297) suggested that the effects of increased adiposity are dependent on genetic background and mechanism of colitis induction. In their SMAD3^-/-^ (129-Smad3tm1Par/J) mouse model, HFD-induced obesity had no effect on *Helicobacter hepaticus*, colitis severity, whereas mice with low caloric intake experience 40% mortality due to infection (297).

#### Leptin

Leptin is a satiety hormone that regulates energy balance as a long-term regulator suppressing food intake preventing obesity. The role of leptin in IBD has been studied, but the results are conflicting and further investigation is required (298–300). Leptin promotes autoimmune diseases and its blockage is protective (pegylated leptin antagonist mutant L39A/D40A/F41A; PEG-MLA) (300). Recent studies with IL10^-/-^ mice showed that the blockage of leptin prevents chronic spontaneous colonic inflammation (300). Although earlier studies in IL-10 mice having also a KO mutation in the leptin gene showed not to have inflammation prevention, compared to the single IL-10 KO deficiency (301). However, in IL-10 KO mice, the deletion of leptin resulted in increased apoptosis of lymphocytes in the lamina propria (301), which supports the hypothesis that inflammatory cells benefit and even survive longer in the presence of leptin ligands, promoting IL-10-driven chronic inflammation. It is possible that this effect supports the hypothesis that a feedback loop cycle could locally exist where a progressive state of intestinal inflammation leads to accumulated gut-mesenteric fat (creeping fat), which (e.g., adipocytes) could produce leptin, positively reinforcing a vicious inflammatory cycle locally.

Part of the modulating effect of leptin on inflammation has been supported by the presence of leptin receptors (LR mRNA gene expression detected) on hematopoietic cells, T-cells and lymphocytes (300), and in the gastric mucosa, where it can also be produced. The main producing tissue is the adipose tissue which increases with obesity. In patients with CD, VAT/FM ratio was associated with leptin and IL-6 concentrations, and higher in short-term than in long-term remission (289). Leptin also increases as a result of positive feedback from TNFα, and it has been proposed that this mechanism may be relevant in early inflammatory stages (302, 303).

The mechanism of how dietary fats may modulate inflammation in IBD could be directly dependent on the accumulation of adipose tissue, or indirectly/alternatively could be due to the effect of fats on gastric physiology, because leptin can be also produced by the gastric mucosa using a seemingly unique 19KD precursor molecule which is distinct to what occurs in the adipose tissue (300). In the stomach, leptin increases its levels according to the feeding regime, which in turn, modifies systemic circulating levels, which could reach cells in inflammatory sites to promote inflammation in cells if they are activated and primed with LR. Fasting and starvation are associated with high local levels of gastrin; but after a meal, leptin locally decreases but increases systemically. Additionally, it has been shown that slow gastric emptying can increase leptin levels in the stomach. Considering that dietary fats influence gastric emptying rates, it is possible that slow gastric emptying caused by a HFD influences leptin levels.

Most recently, differently expressed colonic genes from C57BL/6 mice in response to a HFD (45% EAF vs. normal diet, 15% EAF) have been reported. Of the 21 identified leptin-associated genes found to have an inverse relationship between the two dietary types (HFD, normal fat) *Peli3, Creb1, Enpp2* and *Centg1*, four genes previously reported to play a role in obesity and colon-related diseases, were found to have either a positive or negative relationship between serum leptin or insulin concentration and consumption of either HFD or normal diet (304).

#### Ghrelin

It is known that bacterial lipopolysaccharides mediate diarrhea induced by bacterial infection in the gut (305). In ghrelin-treated mice, this endotoxinemia-induced dysmotility was improved, mainly *via* down-regulation of nitric oxide pathways in the gut (306), decreased production of pro-inflammatory cytokines IL-1β and TNFα, with concomitant increase in anti-inflammatory IL-10 (305, 307). The role of ghrelin in IBD is thought to be attributed to its antagonistic effect on leptin, although several *in vivo* and *in vitro* studies have described both pro- and anti-inflammatory effects from ghrelin (305).

In CD-IBD, Zhao et al. (308) reported upregulation of ghrelin and ghrelin receptor mRNA *via* NF-kB pathway activation and induction of IL-8 production in TNBS-colitis mice. By comparison, Gonzalez-Rey et al. (309) found ghrelin ameliorated severity of TNBS-colitis and suppressed IL-10 levels. Konturek et al. (310) also found accelerated colonic lesion healing in ghrelin treated TNBS-colitis *via* increased nitric oxide and PGE2 release. In UC-IBD, De Smet et al. (311) found that a lack of ghrelin delayed neutrophil infiltration and decreased disease activity index in a model of chronic DSS-colitis, whereas in *C.rodentium*-induced colitis (312) the late stages of infection were associated with increased ghrelin expression, with *in vitro* studies showing ghrelin induced marked proliferation of neurons.

#### Substance P and Obesity

Intracolonic administration of TNBS-colitis has been shown to cause severe acute colitis and changes in the mesenteric and epididymal fat depots arguably described as resemblants of changes in CD with increased pro-inflammatory mediators in these fat depots, including substance P (SP) (2, 12, 313, 314). Such findings indicate that human mesenteric pre-adipocytes contain functional substance P receptors that are linked to pro-inflammatory pathways, and that substance P can directly increase NK-1R expression. Thus, it is possible that mesenteric fat depots may participate in intestinal inflammatory responses *via* substance P-NK-1R-related pathways, as well as other systemic responses to the presence of an ongoing inflammation of the colon.

## Discussion

Herein, we review the evidence on the role of HFDs on the severity of experimental ileitis and colitis in laboratory rodents to further advance our mechanistic understanding of the effects of FAs on intestinal inflammation. While studies conducted directly in humans provide prevalence, incidence and clinical estimates, studies using laboratory rodents performed under controlled conditions allow for mechanistic insights relevant to IBD. However, our review highlights considerable variability in findings between studies. Such discrepancies appear to be dependent on various factors including the rodent model of IBD used (including colitis induction method), feeding trial duration, amount/dosage/source and structural composition of the FA tested, as well as how other factors such as diet compounds, lifestyle, drugs, probiotics and the gut microbiota could interact with the FA to modulate disease. Whereas FA-mediated regulation of pro- and anti-inflammatory T-cell responses *in vivo* remains a largely nascent field, fundamental questions remain concerning FA uptake, intracellular transport and regulatory function. Existing studies give cause for optimism that understanding the molecular interplay between FAs and T-cells will reveal biologically novel and translationally-relevant insights toward the treatment of human diseases.

Despite the great advancement, a limitation to note is that while studies investigating a ‘HFD’ generate relevant data, many do not report in detail the nutritional composition of the diet, particularly the FA profile, and other husbandry factors (as recently discussed) (315), making such studies less reproducible. This is important considering that not only the amount by the type and structure of the FA can influence phenotypic outcomes of disease. In the future it is imperative to improve reporting and to conduct experiments that correlate the mechanisms described, the genetics of the host and the microbiome of the host refining methodological/the designs and the use of germ-free systems or *in vitro/ex vivo* organoids could help further elucidate these mechanisms.

## Author Contributions

AR-P, AB, SI, AT, FC: study design. AB, CC, FS, AT, AG-N: literature review. AB, CC, AR-P, FS: manuscript writing. AB, CC, FS, AG-N, AT, IB, SI, MS, FC, AR-P: review, comments, and editing of final manuscript. All authors contributed to the article and approved the submitted version.

## Funding

Research reported in this publication was supported by the NIH grant DK055812, DK091222 and DK097948 (to FC), T32DK083251 and F32DK117585 (to AB), and P01DK091222 Germ-free and Gut Microbiome Core and R21DK118373 (to AR-P). We acknowledge the Biorepository Core of the NIH Silvio O. Conte Cleveland Digestive Disease Research Core Center (P30DK097948). Partial support also originated for AR-P from a career development award from the Crohn’s and Colitis Foundation.

## Conflict of Interest

The authors declare that the research was conducted in the absence of any commercial or financial relationships that could be construed as a potential conflict of interest.

## Abbreviations

AA, arachidonic acid; AdipoR1, adiponectin receptor 1; Ahr, aryl hydrocarbon receptor; ALA, alpha linoleic acid; Akt, Protein kinase B; CD, Crohn’s disease; Cideb, cell death–inducing DFF45-like effector b; COX, cyclooxygenase; CLA, conjugated linoleic acid; DHA, docosahexaenoic acid; DSS, dextran sodium sulfate; DPA, docosapentaenoic acid; EAF, energy as fat; EPA, eicosapentaenoic acid; ER, endoplasmic reticulum; FA, fatty acid; GSH, glutathione; GPR120, G protein-coupled receptor 120; HDACS, histone deacetylase; HFD, high-fat diet; IBD, inflammatory bowel disease; IEL, intraepithelial lymphocytes; KO, knockout; LA, linoleic acid; LCFA, long-chain fatty acids; LT, leukotrienes; LPS, lipopolysaccharide; MCFA, medium-chain fatty acids; MCP1, monocyte chemoattractant protein 1; MCT, medium-chain triglycerides; MPO, myeloperoxidase activity; MUFA, monounsaturated fatty acid; NFAT, nuclear factor of activated T-cells; n-3, omega-3; n-6, omega-6; PPAR, peroxisome proliferator-activated receptor; PGE2, prostaglandin E2; PUFA, polyunsaturated fatty acid; ROS, reactive oxygen species; SCFA, short-chain fatty acids; Treg, T regulatory; Tconv, conventional T cell; TNBS, 2,4,6-trinitrobenzenesulfonic acid; TLR, toll-like receptor; UC, ulcerative colitis; VDR, vitamin D receptor; VLCFA, very long chain fatty acids.

## References

[1] Boutros M Maron D Inflammatory bowel disease in the obese patient. Clin Colon Rectal Surg (2011) 24:244–52. 10.1055/s-0031-1295687 PMC3311491 23204939

[2] Karagiannides I Pothoulakis C Substance P, obesity, and gut inflammation. Curr Opin Endocrinol Diabetes Obes (2009) 16:47–52. 10.1097/MED.0b013e328321306c 19104238 PMC4404028

[3] Hou JK Abraham B El-Serag H Dietary intake and risk of developing inflammatory bowel disease: a systematic review of the literature. Am J Gastroenterol (2011) 106:563–73. 10.1038/ajg.2011.44 21468064

[4] Martinez-Medina M Denizot J Dreux N Robin F Billard E Bonnet R Western diet induces dysbiosis with increased E coli in CEABAC10 mice, alters host barrier function favouring AIEC colonisation. Gut (2014) 63:116–24. 10.1136/gutjnl-2012-304119 23598352

[5] Bosco N Brahmbhatt V Oliveira M Martin FP Lichti P Raymond F Effects of increase in fish oil intake on intestinal eicosanoids and inflammation in a mouse model of colitis. Lipids Health Dis (2013) 12:81. 10.1186/1476-511X-12-81 23725086 PMC3691874

[6] Ananthakrishnan AN Khalili H Konijeti GG Higuchi LM de Silva P Fuchs CS Long-term intake of dietary fat and risk of ulcerative colitis and Crohn’s disease. Gut (2014) 63:776–84. 10.1136/gutjnl-2013-305304 PMC3915038 23828881

[7] Geerling BJ Dagnelie PC Badart-Smook A Russel MG Stockbrügger RW Brummer RJ Diet as a risk factor for the development of ulcerative colitis. Am J Gastroenterol (2000) 95:1008–13. 10.1111/j.1572-0241.2000.01942.x 10763951

[8] Legaki E Gazouli M Influence of environmental factors in the development of inflammatory bowel diseases. World J Gastrointest Pharmacol Ther (2016) 7:112–25. 10.4292/wjgpt.v7.i1.112 PMC4734944 26855817

[9] Shoda R Matsueda K Yamato S Umeda N Epidemiologic analysis of Crohn disease in Japan: increased dietary intake of n-6 polyunsaturated fatty acids and animal protein relates to the increased incidence of Crohn disease in Japan. Am J Clin Nutr (1996) 63:741–5. 10.1093/ajcn/63.5.741 8615358

[10] Monaco G van Dam S Casal Novo Ribeiro JL Larbi A de Magalhaes JP A comparison of human and mouse gene co-expression networks reveals conservation and divergence at the tissue, pathway and disease levels. BMC Evol Biol (2015) 15:259. 10.1186/s12862-015-0534-7 26589719 PMC4654840

[11] Liu WX Wang T Zhou F Wang Y Xing JW Zhang S Voluntary exercise prevents colonic inflammation in high-fat diet-induced obese mice by up-regulating PPAR-γ activity. Biochem Biophys Res Commun (2015) 459:475–80. 10.1016/j.bbrc.2015.02.047 25701789

[12] Sideri A Stavrakis D Bowe C Shih DQ Fleshner P Arsenescu V Effects of obesity on severity of colitis and cytokine expression in mouse mesenteric fat. Potential role of adiponectin receptor 1. Am J Physiol Gastrointest Liver Physiol (2015) 308:G591–604. 10.1152/ajpgi.00269.2014 PMC4385897 25591865

[13] Marton LT Goulart RA Carvalho ACA Barbalho SM Omega Fatty Acids and Inflammatory Bowel Diseases: An Overview. Int J Mol Sci (2019) 20:1–16. 10.3390/ijms20194851 PMC6801729 31574900

[14] Cariello M Contursi A Gadaleta RM Piccinin E De Santis S Piglionica M Extra-Virgin Olive Oil from Apulian Cultivars and Intestinal Inflammation. Nutrients (2020) 12(4):1084. 10.3390/nu12041084 PMC7230776 32295122

[15] Tou JC Jaczynski J Chen YC Krill for human consumption: nutritional value and potential health benefits. Nutr Rev (2007) 65:63–77. 10.1111/j.1753-4887.2007.tb00283.x 17345959

[16] Grimstad T Bjorndal B Cacabelos D Aasprong OG Janssen EA Omdal R Dietary supplementation of krill oil attenuates inflammation and oxidative stress in experimental ulcerative colitis in rats. Scand J Gastroenterol (2012) 47:49–58. 10.3109/00365521.2011.634025 22126533

[17] de Carvalho C Caramujo MJ The Various Roles of Fatty Acids. Molecules (2018) 23:1–36. 10.3390/molecules23102583 PMC6222795 30304860

[18] Ananthakrishnan AN Khalili H Konijeti GG Higuchi LM De Silva P Korzenik JR A Prospective Study of Long-term Intake of Dietary Fiber and Risk of Crohn’s Disease and Ulcerative Colitis. Gastroenterology (2013) 145:970–7. 10.1053/j.gastro.2013.07.050 PMC3805714 23912083

[19] Rezanka T Very-long-chain fatty acids from the animal and plant kingdoms. Prog Lipid Res (1989) 28:147–87. 10.1016/0163-7827(89)90011-8 2694175

[20] Kris-Etherton PM AHA Science Advisory. Monounsaturated fatty acids and risk of cardiovascular disease. American Heart Association. Nutrition Committee. Circulation (1999) 100:1253–8. 10.1161/01.cir.100.11.1253 10484550

[21] Wen J Khan I Li A Chen X Yang P Song P Alpha-linolenic acid given as an anti-inflammatory agent in a mouse model of colonic inflammation. Food Sci Nutr (2019) 7:3873–82. 10.1002/fsn3.1225 PMC6924294 31890165

[22] Bassaganya-Riera J Hontecillas R Dietary conjugated linoleic acid and n-3 polyunsaturated fatty acids in inflammatory bowel disease. Curr Opin Clin Nutr Metab Care (2010) 13:569–73. 10.1097/MCO.0b013e32833b648e PMC2947030 20508519

[23] St-Onge MP Jones PJ Physiological effects of medium-chain triglycerides: potential agents in the prevention of obesity. J Nutr (2002) 132:329–32. 10.1093/jn/132.3.329 11880549

[24] Scorletti E Byrne CD Omega-3 fatty acids, hepatic lipid metabolism, and nonalcoholic fatty liver disease. Annu Rev Nutr (2013) 33:231–48. 10.1146/annurev-nutr-071812-161230 23862644

[25] Johnson M Bradford C Omega-3, Omega-6 and Omega-9 Fatty Acids: Implications for Cardiovascular and Other Diseases. J Glycom Lipidomics (2014) 4:1–8. 0.4172/2153-0637.1000123

[26] Lowry RR Tinsley IJ Oleic and linoleic acid interaction in polyunsaturated fatty acid metabolism in the rat. J Nutr (1966) 88:26–32. 10.1093/jn/88.1.26 5900628

[27] Abdolmaleki F Kovanen PT Mardani R Gheibi-Hayat SM Bo S Sahebkar A Resolvins: Emerging Players in Autoimmune and Inflammatory Diseases. Clin Rev Allergy Immunol (2020) 58:82–91. 10.1007/s12016-019-08754-9 31267470

[28] Duvall MG Levy BD DHA- and EPA-derived resolvins, protectins, and maresins in airway inflammation. Eur J Pharmacol (2016) 785:144–55. 10.1016/j.ejphar.2015.11.001 PMC4854800 26546247

[29] de Silva PS Olsen A Christensen J Schmidt EB Overvaad K Tjonneland A An association between dietary arachidonic acid, measured in adipose tissue, and ulcerative colitis. Gastroenterology (2010) 139:1912–7. 10.1053/j.gastro.2010.07.065 20950616

[30] Nishida T Miwa H Shigematsu A Yamamoto M Iida M Fujishima M Increased arachidonic acid composition of phospholipids in colonic mucosa from patients with active ulcerative colitis. Gut (1987) 28:1002–7. 10.1136/gut.28.8.1002 PMC1433134 3117625

[31] Ungaro F Rubbino F Danese S D’Alessio S Actors and Factors in the Resolution of Intestinal Inflammation: Lipid Mediators As a New Approach to Therapy in Inflammatory Bowel Diseases. Front Immunol (2017) 8:1331. 10.3389/fimmu.2017.01331 29109724 PMC5660440

[32] Yoon BK Jackman JA Valle-Gonzalez ER Cho NJ Antibacterial Free Fatty Acids and Monoglycerides: Biological Activities, Experimental Testing, and Therapeutic Applications. Int J Mol Sci (2018) 19:1–40. 10.3390/ijms19041114 PMC5979495 29642500

[33] Mañé J Pedrosa E Lorén V Ojanguren I Fluvià L Cabré E Partial replacement of dietary (n-6) fatty acids with medium-chain triglycerides decreases the incidence of spontaneous colitis in interleukin-10-deficient mice. J Nutr (2009) 139:603–10. 10.3945/jn.108.101170 19126671

[34] Kono H Fujii H Ogiku M Tsuchiya M Ishii K Hara M Enteral diets enriched with medium-chain triglycerides and N-3 fatty acids prevent chemically induced experimental colitis in rats. Transl Res (2010) 156:282–91. 10.1016/j.trsl.2010.07.012 20970751

[35] Ohta N Tsujikawa T Nakamura T Andoh A Sasaki M Bamba T A comparison of the effects of medium- and long-chain triglycerides on neutrophil stimulation in experimental ileitis. J Gastroenterol (2003) 38:127–33. 10.1007/s005350300021 12640525

[36] Laroui H Ingersoll SA Liu HC Baker MT Ayyadurai S Charania MA Dextran sodium sulfate (DSS) induces colitis in mice by forming nano-lipocomplexes with medium-chain-length fatty acids in the colon. PloS One (2012) 7:e32084. 10.1371/journal.pone.0032084 22427817 PMC3302894

[37] Schwingshackl L Strasser B Hoffmann G Effects of monounsaturated fatty acids on cardiovascular risk factors: a systematic review and meta-analysis. Ann Nutr Metab (2011) 59:176–86. 10.1159/000334071 22142965

[38] Gillingham LG Harris-Janz S Jones PJ Dietary monounsaturated fatty acids are protective against metabolic syndrome and cardiovascular disease risk factors. Lipids (2011) 46:209–28. 10.1007/s11745-010-3524-y 21308420

[39] Liu X Kris-Etherton PM West SG Lamarche B Jenkins DJ Fleming JA Effects of canola and high-oleic-acid canola oils on abdominal fat mass in individuals with central obesity. Obes (Silver Spring) (2016) 24:2261–8. 10.1002/oby.21584 PMC5119743 27804268

[40] Cani PD Osto M Geurts L Everard A Involvement of gut microbiota in the development of low-grade inflammation and type 2 diabetes associated with obesity. Gut Microbes (2012) 3:279–88. 10.4161/gmic.19625 PMC3463487 22572877

[41] Blok WL Katan MB van der Meer JW Modulation of inflammation and cytokine production by dietary (n-3) fatty acids. J Nutr (1996) 126:1515–33. 10.1093/jn/126.6.1515 8648424

[42] Hart AR Luben R Olsen A Tjonneland A Linseisen J Nagel G Diet in the aetiology of ulcerative colitis: a European prospective cohort study. Digestion (2008) 77:57–64. 10.1159/000121412 18349539

[43] John S Luben R Shrestha SS Welch A Khaw KT Hart AR Dietary n-3 polyunsaturated fatty acids and the aetiology of ulcerative colitis: a UK prospective cohort study. Eur J Gastroenterol Hepatol (2010) 22:602–6. 10.1097/MEG.0b013e3283352d05 20216220

[44] Hekmatdoost A Mirshafiey A Feizabadi MM Djazayeri A Polyunsaturated fatty acids, microflora and colitis. Ann Nutr Metab (2009) 55:325. 10.1159/000248990 19844088

[45] Tyagi A Kumar U Santosh VS Reddy S Mohammed SB Ibrahim A Partial replacement of dietary linoleic acid with long chain n-3 polyunsaturated fatty acids protects against dextran sulfate sodium-induced colitis in rats. Prostaglandins Leukot Essent Fatty Acids (2014) 91:289–97. 10.1016/j.plefa.2014.09.003 25451558

[46] Bertevello PL De Nardi L Torrinhas RS Logullo AF Waitzberg DL Partial replacement of omega-6 fatty acids with medium-chain triglycerides, but not olive oil, improves colon cytokine response and damage in experimental colitis. JPEN J Parenter Enteral Nutr (2012) 36:442–8. 10.1177/0148607111421788 22269895

[47] Campos FG Waitzberg DL Habr-Gama A Logullo AF Noronha IL Jancar S Impact of parenteral n-3 fatty acids on experimental acute colitis. Br J Nutr (2002) 87 Suppl 1:S83–88. 10.1079/bjn2001460 11898774

[48] Maattanen P Lurz E Botts SR Wu RY Robinson SC Yeung CW Plant- and Fish-Derived n-3 PUFAs Suppress Citrobacter Rodentium-Induced Colonic Inflammation. Mol Nutr Food Res (2020) 64:e1900873. 10.1002/mnfr.201900873 31945799

[49] Baker EJ Miles EA Burdge GC Yaqoob P Calder PC Metabolism and functional effects of plant-derived omega-3 fatty acids in humans. Prog Lipid Res (2016) 64:30–56. 10.1016/j.plipres.2016.07.002 27496755

[50] Yao J Lu Y Zhi M Hu P Wu W Gao X Dietary n3 polyunsaturated fatty acids ameliorate Crohn’s disease in rats by modulating the expression of PPARgamma/NFAT. Mol Med Rep (2017) 16:8315–22. 10.3892/mmr.2017.7673 28990050

[51] Andoh A Tsujikawa T Ishizuka I Araki Y Sasaki M Koyama S N-3 fatty acid-rich diet prevents early response of interleukin-6 elevation in trinitrobenzene sulfonic acid-induced enteritis. Int J Mol Med (2003) 12:721–5. 10.3892/ijmm.12.5.721 14533000

[52] Matsunaga H Hokari R Kurihara C Okada Y Takebayashi K Okudaira K Omega-3 fatty acids exacerbate DSS-induced colitis through decreased adiponectin in colonic subepithelial myofibroblasts. Inflammation Bowel Dis (2008) 14:1348–57. 10.1002/ibd.20491 18484673

[53] Mbodji K Charpentier C Guerin C Querec C Bole-Feysot C Aziz M Adjunct therapy of n-3 fatty acids to 5-ASA ameliorates inflammatory score and decreases NF-kappaB in rats with TNBS-induced colitis. J Nutr Biochem (2013) 24:700–5. 10.1016/j.jnutbio.2012.03.022 22841543

[54] Hokari R Matsunaga H Miura S Effect of dietary fat on intestinal inflammatory diseases. J Gastroenterol Hepatol (2013) 28 Suppl 4:33–6. 10.1111/jgh.12252 24251701

[55] Matsunaga H Hokari R Kurihara C Okada Y Takebayashi K Okudaira K Omega-3 polyunsaturated fatty acids ameliorate the severity of ileitis in the senescence accelerated mice (SAM)P1/Yit mice model. Clin Exp Immunol (2009) 158:325–33. 10.1111/j.1365-2249.2009.04020.x PMC2792829 19793338

[56] Ergas D Eliat S Mendlovic Z Sthoeger M N-3 Fatty Acids and the Immune System in Autoimmunity. Isr Med Assoc J (2002) 4:3510–6. 11802309

[57] Perel P Roberts I Sena E Wheble P Briscoe C Sandercock P Comparison of treatment effects between animal experiments and clinical trials: systematic review. BMJ (2007) 334:197. 10.1136/bmj.39048.407928.BE 17175568 PMC1781970

[58] Hackam DG Redelmeier DA Translation of research evidence from animals to humans. JAMA (2006) 296:1731–2. 10.1001/jama.296.14.1731 17032985

[59] Zhu L Shi T Zhong C Wang Y Chang M Liu X IL-10 and IL-10 Receptor Mutations in Very Early Onset Inflammatory Bowel Disease. Gastroenterol Res (2017) 10:65–9. 10.14740/gr740w PMC5412537 28496525

[60] Bielohuby M Menhofer D Kirchner H Stoehr BJ Muller TD Stock P Induction of ketosis in rats fed low-carbohydrate, high-fat diets depends on the relative abundance of dietary fat and protein. Am J Physiol Endocrinol Metab (2011) 300:E65–76. 10.1152/ajpendo.00478.2010 20943751

[61] Takahashi M Ikemoto S Ezaki O Effect of the fat/carbohydrate ratio in the diet on obesity and oral glucose tolerance in C57BL/6J mice. J Nutr Sci Vitaminol (Tokyo) (1999) 45:583–93. 10.3177/jnsv.45.583 10683810

[62] Speakman JR Use of high-fat diets to study rodent obesity as a model of human obesity. Int J Obes (Lond) (2019) 43:1491–2. 10.1038/s41366-019-0363-7 30967607

[63] Lassenius M I Pietilainen KH Kaartinen K Pussinen PJ Syrjanen J Forsblom C Bacterial endotoxin activity in human serum is associated with dyslipidemia, insulin resistance, obesity, and chronic inflammation. Diabetes Care (2011) 34:1809–15. 10.2337/dc10-2197 PMC3142060 21636801

[64] Gulhane M Murray L Lourie R Tong H Sheng YH Wang R High Fat Diets Induce Colonic Epithelial Cell Stress and Inflammation that is Reversed by IL-22. Sci Rep (2016) 6:28990. 10.1038/srep28990 27350069 PMC4924095

[65] Ding S Chi MM Scull BP Rigby R Schwerbrock NM Magness S High-fat diet: bacteria interactions promote intestinal inflammation which precedes and correlates with obesity and insulin resistance in mouse. PloS One (2010) 5:e12191. 10.1371/journal.pone.0012191 20808947 PMC2922379

[66] Kim KA Gu W Lee IA Joh EH Kim DH High fat diet-induced gut microbiota exacerbates inflammation and obesity in mice via the TLR4 signaling pathway. PloS One (2012) 7:e47713. 10.1371/journal.pone.0047713 23091640 PMC3473013

[67] Liu Z Brooks RS Ciappio ED Kim SJ Crott JW Bennett G Diet-induced obesity elevates colonic TNF-alpha in mice and is accompanied by an activation of Wnt signaling: a mechanism for obesity-associated colorectal cancer. J Nutr Biochem (2012) 23:1207–13. 10.1016/j.jnutbio.2011.07.002 PMC4142203 22209007

[68] Luck H Tsai S Chung J Clemente-Casares X Ghazarian M Revelo XS Regulation of obesity-related insulin resistance with gut anti-inflammatory agents. Cell Metab (2015) 21:527–42. 10.1016/j.cmet.2015.03.001 25863246

[69] Cani PD Bibiloni R Knauf C Waget A Neyrinck AM Delzenne NM Changes in gut microbiota control metabolic endotoxemia-induced inflammation in high-fat diet-induced obesity and diabetes in mice. Diabetes (2008) 57:1470–81. 10.2337/db07-1403 18305141

[70] Hildebrandt MA Hoffmann C Sherrill-Mix SA Keilbaugh SA Hamady M Chen YY High-fat diet determines the composition of the murine gut microbiome independently of obesity. Gastroenterology (2009) 137:1716–1724 e1711-1712. 10.1053/j.gastro.2009.08.042 19706296 PMC2770164

[71] Serino M Luche E Gres S Baylac A Berge M Cenac C Metabolic adaptation to a high-fat diet is associated with a change in the gut microbiota. Gut (2012) 61:543–53. 10.1136/gutjnl-2011-301012 PMC3292714 22110050

[72] Gruber L Kisling S Lichti P Martin FP May S Klingenspor M High fat diet accelerates pathogenesis of murine Crohn’s disease-like ileitis independently of obesity. PloS One (2013) 8:e71661. 10.1371/journal.pone.0071661 23977107 PMC3745443

[73] Neurath MF Fuss I Kelsall BL Stuber E Strober W Antibodies to interleukin 12 abrogate established experimental colitis in mice. J Exp Med (1995) 182:1281–90. 10.1084/jem.182.5.1281 PMC2192205 7595199

[74] van der Logt EM Blokzijl T van der Meer R Faber KN Dijkstra G Westernized high-fat diet accelerates weight loss in dextran sulfate sodium-induced colitis in mice, which is further aggravated by supplementation of heme. J Nutr Biochem (2013) 24:1159–65. 10.1016/j.jnutbio.2012.09.001 23246033

[75] Cheng L Jin H Qiang Y Wu S Yan C Han M High fat diet exacerbates dextran sulfate sodium induced colitis through disturbing mucosal dendritic cell homeostasis. Int Immunopharmacol (2016) 40:1–10. 10.1016/j.intimp.2016.08.018 27567245

[76] Kim IW Myung SJ Do MY Ryu YM Kim MJ Do EJ Western-style diets induce macrophage infiltration and contribute to colitis-associated carcinogenesis. J Gastroenterol Hepatol (2010) 25:1785–94. 10.1111/j.1440-1746.2010.06332.x 21039842

[77] Okada Y Tsuzuki Y Sato H Narimatsu K Hokari R Kurihara C Trans fatty acids exacerbate dextran sodium sulphate-induced colitis by promoting the up-regulation of macrophage-derived proinflammatory cytokines involved in T helper 17 cell polarization. Clin Exp Immunol (2013) 174:459–71. 10.1111/cei.12200 PMC3826312 24028683

[78] Li X Wei X Sun Y Du J Li X Xun Z High-fat diet promotes experimental colitis by inducing oxidative stress in the colon. Am J Physiol Gastrointest Liver Physiol (2019) 317:G453–62. 10.1152/ajpgi.00103.2019 31411504

[79] Lee JC Lee HY Kim TK Kim MS Park YM Kim J Obesogenic diet-induced gut barrier dysfunction and pathobiont expansion aggravate experimental colitis. PloS One (2017) 12:e0187515. 10.1371/journal.pone.0187515 29107964 PMC5673181

[80] Lu P Bar-Yoseph F Levi L Lifshitz Y Witte-Bouma J de Bruijn AC High beta-palmitate fat controls the intestinal inflammatory response and limits intestinal damage in mucin Muc2 deficient mice. PloS One (2013) 8:e65878. 10.1371/journal.pone.0065878 23776564 PMC3680492

[81] Paik J Fierce Y Treuting PM Brabb T Maggio-Price L High-fat diet-induced obesity exacerbates inflammatory bowel disease in genetically susceptible Mdr1a-/- male mice. J Nutr (2013) 143:1240–7. 10.3945/jn.113.174615 23761644

[82] Mi Y Chin YX Cao WX Chang YG Lim PE Xue CH Native kappa-carrageenan induced-colitis is related to host intestinal microecology. Int J Biol Macromol (2020) 147:284–94. 10.1016/j.ijbiomac.2020.01.072 31926226

[83] Lee JY Cevallos SA Byndloss MX Tiffany CR Olsan EE Butler BP High-Fat Diet and Antibiotics Cooperatively Impair Mitochondrial Bioenergetics to Trigger Dysbiosis that Exacerbates Pre-inflammatory Bowel Disease. Cell Host Microbe (2020) 28:273–84.e276. 10.1016/j.chom.2020.06.001 32668218 PMC7429289

[84] Mazur-Bialy A I Bilski J Wojcik D Brzozowski B Surmiak M Hubalewska-Mazgaj M Beneficial Effect of Voluntary Exercise on Experimental Colitis in Mice Fed a High-Fat Diet: The Role of Irisin, Adiponectin and Proinflammatory Biomarkers. Nutrients (2017) 9(4):410. 10.3390/nu9040410 PMC5409749 28425943

[85] Jang HM Han SK Kim JK Oh SJ Jang HB Kim DH Lactobacillus sakei Alleviates High-Fat-Diet-Induced Obesity and Anxiety in Mice by Inducing AMPK Activation and SIRT1 Expression and Inhibiting Gut Microbiota-Mediated NF-kappaB Activation. Mol Nutr Food Res (2019) 63:e1800978. 10.1002/mnfr.201800978 30636176

[86] Jang SE Min SW Lactobacillus sakei S1 Improves Colitis Induced by 2,4,6-Trinitrobenzene Sulfonic Acid by the Inhibition of NF-kappaB Signaling in Mice. J Microbiol Biotechnol (2020) 30:71–8. 10.4014/jmb.1907.07050 PMC9728177 31635441

[87] Kim H II Yun SW Han MJ Jang SE Kim DH IL-10 Expression-Inducing Gut Bacteria Alleviate High-Fat Diet-Induced Obesity and Hyperlipidemia in Mice. J Microbiol Biotechnol (2020) 30:599–603. 10.4014/jmb.1912.12014 31986244 PMC9728323

[88] Wang X Yang Z Xu X Jiang H Cai C Yu G Odd-numbered agaro-oligosaccharides alleviate type 2 diabetes mellitus and related colonic microbiota dysbiosis in mice. Carbohydr Polym (2020) 240:116261. 10.1016/j.carbpol.2020.116261 32475553

[89] Penkava RR Poellein S Rothenberg J Fine-needle aspiration biopsy with CT guidance. Am Fam Physician (1981) 24:155–60. 7258062

[90] Enos RT Davis JM Velázquez KT McClellan JL Day SD Carnevale KA Influence of dietary saturated fat content on adiposity, macrophage behavior, inflammation, and metabolism: composition matters. J Lipid Res (2013) 54:152–63. 10.1194/jlr.M030700 PMC3520521 23103474

[91] Maattanen P Lurz E Botts SR Wu RY Yeung CW Li B Ground flaxseed reverses protection of a reduced-fat diet against Citrobacter rodentium-induced colitis. Am J Physiol Gastrointest Liver Physiol (2018) 315:G788–98. 10.1152/ajpgi.00101.2018 30095298

[92] Singh KK Mridula D Rehal J Barnwal P Flaxseed: a potential source of food, feed and fiber. Crit Rev Food Sci Nutr (2011) 51:210–22. 10.1080/10408390903537241 21390942

[93] Zarepoor L Lu JT Zhang C Wu W Lepp D Robinson L Dietary flaxseed intake exacerbates acute colonic mucosal injury and inflammation induced by dextran sodium sulfate. Am J Physiol Gastrointest Liver Physiol (2014) 306:G1042–55. 10.1152/ajpgi.00253.2013 24763556

[94] Power KA Lepp D Zarepoor L Monk JM Wu W Tsao R Dietary flaxseed modulates the colonic microenvironment in healthy C57Bl/6 male mice which may alter susceptibility to gut-associated diseases. J Nutr Biochem (2016) 28:61–9. 10.1016/j.jnutbio.2015.09.028 26878783

[95] Ericsson AC Davis JW Spollen W Bivens N Givan S Hagan CE Effects of vendor and genetic background on the composition of the fecal microbiota of inbred mice. PloS One (2015) 10:e0116704. 10.1371/journal.pone.0116704 25675094 PMC4326421

[96] Franklin CL Ericsson AC Microbiota and reproducibility of rodent models. Lab Anim (2017) 46:114–22. 10.1038/laban.1222 PMC5762113 28328896

[97] Cohen SL Moore AM Ward WE Flaxseed oil and inflammation-associated bone abnormalities in interleukin-10 knockout mice. J Nutr Biochem (2005) 16:368–74. 10.1016/j.jnutbio.2005.01.008 15936649

[98] Periasamy S Hsu DZ Chandrasekaran VR Liu MY Sesame oil accelerates healing of 2,4,6-trinitrobenzenesulfonic acid-induced acute colitis by attenuating inflammation and fibrosis. JPEN J Parenter Enteral Nutr (2013) 37:674–82. 10.1177/0148607112468768 23243149

[99] Kondamudi PK Kovelamudi H Mathew G Nayak PG Rao MC Shenoy RR Investigation of sesamol on myeloperoxidase and colon morphology in acetic acid-induced inflammatory bowel disorder in albino rats. ScientificWorldJournal (2014) 2014:802701. 10.1155/2014/802701 24616646 PMC3926374

[100] Tateishi N Kakutani S Kawashima H Shibata H Morita I Dietary supplementation of arachidonic acid increases arachidonic acid and lipoxin A(4) contents in colon, but does not affect severity or prostaglandin E(2) content in murine colitis model. Lipids Health Dis (2014) 13:30. 10.1186/1476-511X-13-30 24507383 PMC3928921

[101] Gurzell EA Wiesinger JA Morkam C Hemmrich S Harris WS Fenton J I Is the omega-3 index a valid marker of intestinal membrane phospholipid EPA+DHA content? Prostaglandins Leukot Essent Fatty Acids (2014) 91:87–96. 10.1016/j.plefa.2014.04.001 24913088 PMC4127132

[102] Ye J Li JZ Liu Y Li X Yang T Ma X Cideb, an ER- and lipid droplet-associated protein, mediates VLDL lipidation and maturation by interacting with apolipoprotein B. Cell Metab (2009) 9:177–90. 10.1016/j.cmet.2008.12.013 19187774

[103] Zhang LJ Wang C Yuan Y Wang H Wu J Liu F Cideb facilitates the lipidation of chylomicrons in the small intestine. J Lipid Res (2014) 55:1279–87. 10.1194/jlr.M046482 PMC4076075 24831470

[104] Sun C Zhao Y Gao X Yuan Y Wang C Wang Y Cideb Deficiency Aggravates Dextran Sulfate Sodium-induced Ulcerative Colitis in Mice by Exacerbating the Oxidative Burden in Colonic Mucosa. Inflammation Bowel Dis (2017) 23:1338–47. 10.1097/MIB.0000000000001196 28719542

[105] Fuhrer A Sprenger N Kurakevich E Borsig L Chassard C Hennet T Milk sialyllactose influences colitis in mice through selective intestinal bacterial colonization. J Exp Med (2010) 207:2843–54. 10.1084/jem.20101098 PMC3005226 21098096

[106] Knoch B Barnett MP Cooney J McNabb WC Barraclough D Laing W Dietary oleic acid as a control fatty acid for polyunsaturated fatty acid intervention studies: a transcriptomics and proteomics investigation using interleukin-10 gene-deficient mice. Biotechnol J (2010) 5:1226–40. 10.1002/biot.201000066 20872728

[107] Wunderlich CM Ackermann PJ Ostermann AL Adams-Quack P Vogt MC Tran ML Obesity exacerbates colitis-associated cancer via IL-6-regulated macrophage polarisation and CCL-20/CCR-6-mediated lymphocyte recruitment. Nat Commun (2018) 9:1646. 10.1038/s41467-018-03773-0 29695802 PMC5916940

[108] Hoang-Yen Tran D Hoang-Ngoc Tran D Mattai SA Sallam T Ortiz C Lee EC Cathelicidin suppresses lipid accumulation and hepatic steatosis by inhibition of the CD36 receptor. Int J Obes (Lond) (2016) 40:1424–34. 10.1038/ijo.2016.90 PMC5014693 27163748

[109] Lu Y Li X Liu S Zhang Y Zhang D Toll-like Receptors and Inflammatory Bowel Disease. Front Immunol (2018) 9:72. 10.3389/fimmu.2018.00072 29441063 PMC5797585

[110] Eguchi K Manabe I Oishi-Tanaka Y Ohsugi M Kono N Ogata F Saturated fatty acid and TLR signaling link beta cell dysfunction and islet inflammation. Cell Metab (2012) 15:518–33. 10.1016/j.cmet.2012.01.023 22465073

[111] Yeop Han C Kargi AY Omer M Chan CK Wabitsch M O’Brien KD Differential effect of saturated and unsaturated free fatty acids on the generation of monocyte adhesion and chemotactic factors by adipocytes: dissociation of adipocyte hypertrophy from inflammation. Diabetes (2010) 59:386–96. 10.2337/db09-0925 PMC2809975 19934003

[112] Kim KA Lee IA Gu W Hyam SR Kim DH beta-Sitosterol attenuates high-fat diet-induced intestinal inflammation in mice by inhibiting the binding of lipopolysaccharide to toll-like receptor 4 in the NF-kappaB pathway. Mol Nutr Food Res (2014) 58:963–72. 10.1002/mnfr.201300433 24402767

[113] Charpentier C Chan R Salameh E Mbodji K Ueno A Coeffier M Dietary n-3 PUFA May Attenuate Experimental Colitis. Mediators Inflammation (2018) 2018:8430614. 10.1155/2018/8430614 PMC5833476 29670469

[114] Yamada H Umemoto T Kakei M Momomura S I Kawakami M Ishikawa SE Eicosapentaenoic acid shows anti-inflammatory effect via GPR120 in 3T3-L1 adipocytes and attenuates adipose tissue inflammation in diet-induced obese mice. Nutr Metab (Lond) (2017) 14:33. 10.1186/s12986-017-0188-0 28503189 PMC5422876

[115] Talukdar S Olefsky JM Osborn O Targeting GPR120 and other fatty acid-sensing GPCRs ameliorates insulin resistance and inflammatory diseases. Trends Pharmacol Sci (2011) 32:543–50. 10.1016/j.tips.2011.04.004 PMC3419590 21663979

[116] Hsueh HW Zhou Z Whelan J Allen KG Moustaid-Moussa N Kim H Stearidonic and eicosapentaenoic acids inhibit interleukin-6 expression in ob/ob mouse adipose stem cells via Toll-like receptor-2-mediated pathways. J Nutr (2011) 141:1260–6. 10.3945/jn.110.132571 21562237

[117] Murumalla RK Gunasekaran MK Padhan JK Bencharif K Gence L Festy F Fatty acids do not pay the toll: effect of SFA and PUFA on human adipose tissue and mature adipocytes inflammation. Lipids Health Dis (2012) 11:175. 10.1186/1476-511X-11-175 23259689 PMC3551671

[118] Su CG Wen X Bailey ST Jiang W Rangwala SM Keilbaugh SA A novel therapy for colitis utilizing PPAR-gamma ligands to inhibit the epithelial inflammatory response. J Clin Invest (1999) 104:383–9. 10.1172/JCI7145 PMC408529 10449430

[119] Adachi M Kurotani R Morimura K Shah Y Sanford M Madison BB Peroxisome proliferator activated receptor gamma in colonic epithelial cells protects against experimental inflammatory bowel disease. Gut (2006) 55:1104–13. 10.1136/gut.2005.081745 PMC1513267 16547072

[120] Zeng C Xiao JH Chang MJ Wang JL Beneficial effects of THSG on acetic acid-induced experimental colitis: involvement of upregulation of PPAR-gamma and inhibition of the Nf-Kappab inflammatory pathway. Molecules (2011) 16:8552–68. 10.3390/molecules16108552 PMC6264228 21993246

[121] Ricote M Li AC Willson TM Kelly CJ Glass CK The peroxisome proliferator-activated receptor-gamma is a negative regulator of macrophage activation. Nature (1998) 391:79–82. 10.1038/34178 9422508

[122] Andersen V Christensen J Ernst A Jacobsen BA Tjonneland A Krarup HB Polymorphisms in NF-kappaB, PXR, LXR, PPARgamma and risk of inflammatory bowel disease. World J Gastroenterol (2011) 17:197–206. 10.3748/wjg.v17.i2.197 21245992 PMC3020373

[123] Sugawara K Olson TS Moskaluk CA Stevens BK Hoang S Kozaiwa K Linkage to peroxisome proliferator-activated receptor-gamma in SAMP1/YitFc mice and in human Crohn’s disease. Gastroenterology (2005) 128:351–60. 10.1053/j.gastro.2004.11.001 15685547

[124] Raman P Kaplan BL Thompson JT Vanden Heuvel JP Kaminski NE 15-Deoxy-delta12,14-prostaglandin J2-glycerol ester, a putative metabolite of 2-arachidonyl glycerol, activates peroxisome proliferator activated receptor gamma. Mol Pharmacol (2011) 80:201–9. 10.1124/mol.110.070441 PMC3127542 21511917

[125] Chung SW Kang BY Kim TS Inhibition of interleukin-4 production in CD4+ T cells by peroxisome proliferator-activated receptor-gamma (PPAR-gamma) ligands: involvement of physical association between PPAR-gamma and the nuclear factor of activated T cells transcription factor. Mol Pharmacol (2003) 64:1169–79. 10.1124/mol.64.5.1169 14573767

[126] Wahli W Michalik L PPARs at the crossroads of lipid signaling and inflammation. Trends Endocrinol Metab (2012) 23:351–63. 10.1016/j.tem.2012.05.001 22704720

[127] Hassan A Ibrahim A Mbodji K Coeffier M Ziegler F Bounoure F An alpha-linolenic acid-rich formula reduces oxidative stress and inflammation by regulating NF-kappaB in rats with TNBS-induced colitis. J Nutr (2010) 140:1714–21. 10.3945/jn.109.119768 20724486

[128] Marion-Letellier R Butler M Dechelotte P Playford RJ Ghosh S Comparison of cytokine modulation by natural peroxisome proliferator-activated receptor gamma ligands with synthetic ligands in intestinal-like Caco-2 cells and human dendritic cells–potential for dietary modulation of peroxisome proliferator-activated receptor gamma in intestinal inflammation. Am J Clin Nutr (2008) 87:939–48. 10.1093/ajcn/87.4.939 18400717

[129] Ibrahim A Mbodji K Hassan A Aziz M Boukhettala N Coeffier M Anti-inflammatory and anti-angiogenic effect of long chain n-3 polyunsaturated fatty acids in intestinal microvascular endothelium. Clin Nutr (2011) 30:678–87. 10.1016/j.clnu.2011.05.002 21632157

[130] Kong W Yen JH Vassiliou E Adhikary S Toscano MG Ganea D Docosahexaenoic acid prevents dendritic cell maturation and in vitro and in vivo expression of the IL-12 cytokine family. Lipids Health Dis (2010) 9:12. 10.1186/1476-511X-9-12 20122166 PMC2827414

[131] Kong W Yen JH Ganea D Docosahexaenoic acid prevents dendritic cell maturation, inhibits antigen-specific Th1/Th17 differentiation and suppresses experimental autoimmune encephalomyelitis. Brain Behav Immun (2011) 25:872–82. 10.1016/j.bbi.2010.09.012 PMC3031664 20854895

[132] Loscher CE Draper E Leavy O Kelleher D Mills KH Roche HM Conjugated linoleic acid suppresses NF-kappa B activation and IL-12 production in dendritic cells through ERK-mediated IL-10 induction. J Immunol (2005) 175:4990–8. 10.4049/jimmunol.175.8.4990 16210601

[133] Straus DS Glass CK Anti-inflammatory actions of PPAR ligands: new insights on cellular and molecular mechanisms. Trends Immunol (2007) 28:551–8. 10.1016/j.it.2007.09.003 17981503

[134] Szanto A Nagy L The many faces of PPARgamma: anti-inflammatory by any means? Immunobiology (2008) 213:789–803. 10.1016/j.imbio.2008.07.015 18926294

[135] Varga T Nagy L Nuclear receptors, transcription factors linking lipid metabolism and immunity: the case of peroxisome proliferator-activated receptor gamma. Eur J Clin Invest (2008) 38:695–707. 10.1111/j.1365-2362.2008.02022.x 18837794

[136] de Urquiza AM Liu S Sjoberg M Zetterstrom RH Griffiths W Sjovall J Docosahexaenoic acid, a ligand for the retinoid X receptor in mouse brain. Science (2000) 290:2140–4. 10.1126/science.290.5499.2140 11118147

[137] Lengqvist J Mata De Urquiza A Bergman AC Willson TM Sjovall J Perlmann T Polyunsaturated fatty acids including docosahexaenoic and arachidonic acid bind to the retinoid X receptor alpha ligand-binding domain. Mol Cell Proteomics (2004) 3:692–703. 10.1074/mcp.M400003-MCP200 15073272

[138] Gurzell EA Teague H Harris M Clinthorne J Shaikh SR Fenton J I DHA-enriched fish oil targets B cell lipid microdomains and enhances ex vivo and in vivo B cell function. J Leukoc Biol (2013) 93:463–70. 10.1189/jlb.0812394 PMC3597837 23180828

[139] Braissant O Foufelle F Scotto C Dauca M Wahli W Differential expression of peroxisome proliferator-activated receptors (PPARs): tissue distribution of PPAR-alpha, -beta, and -gamma in the adult rat. Endocrinology (1996) 137:354–66. 10.1210/endo.137.1.8536636 8536636

[140] de Souza CO Vannice GK Rosa Neto JC Calder PC Is Palmitoleic Acid a Plausible Nonpharmacological Strategy to Prevent or Control Chronic Metabolic and Inflammatory Disorders? Mol Nutr Food Res (2018) 62:1–12. 10.1002/mnfr.201700504 28980402

[141] Issemann I Green S Activation of a member of the steroid hormone receptor superfamily by peroxisome proliferators. Nature (1990) 347:645–50. 10.1038/347645a0 2129546

[142] Pall H Zaman MM Andersson C Freedman SD Decreased peroxisome proliferator activated receptor alpha is associated with bile duct injury in cystic fibrosis transmembrane conductance regulator-/- mice. J Pediatr Gastroenterol Nutr (2006) 42:275–81. 10.1097/01.mpg.0000189368.37535.42 16540796

[143] Marin HE Peraza MA Billin AN Willson TM Ward JM Kennett MJ Ligand activation of peroxisome proliferator-activated receptor beta inhibits colon carcinogenesis. Cancer Res (2006) 66:4394–401. 10.1158/0008-5472.CAN-05-4277 16618765

[144] Peters JM Hollingshead HE Gonzalez FJ Role of peroxisome-proliferator-activated receptor beta/delta (PPARbeta/delta) in gastrointestinal tract function and disease. Clin Sci (Lond) (2008) 115:107–27. 10.1042/CS20080022 PMC2705117 18616431

[145] Forman BM Chen J Evans RM Hypolipidemic drugs, polyunsaturated fatty acids, and eicosanoids are ligands for peroxisome proliferator-activated receptors alpha and delta. Proc Natl Acad Sci USA (1997) 94:4312–7. 10.1073/pnas.94.9.4312 PMC20719 9113986

[146] Xu HE Lambert MH Montana VG Parks DJ Blanchard SG Brown PJ Molecular recognition of fatty acids by peroxisome proliferator-activated receptors. Mol Cell (1999) 3:397–403. 10.1016/s1097-2765(00)80467-0 10198642

[147] Welch JS Ricote M Akiyama TE Gonzalez FJ Glass CK PPARgamma and PPARdelta negatively regulate specific subsets of lipopolysaccharide and IFN-gamma target genes in macrophages. Proc Natl Acad Sci USA (2003) 100:6712–7. 10.1073/pnas.1031789100 PMC164512 12740443

[148] Monk JM Kim W Callaway E Turk HF Foreman JE Peters JM Immunomodulatory action of dietary fish oil and targeted deletion of intestinal epithelial cell PPARdelta in inflammation-induced colon carcinogenesis. Am J Physiol Gastrointest Liver Physiol (2012) 302:G153–167. 10.1152/ajpgi.00315.2011 PMC3345959 21940900

[149] Hollingshead HE Morimura K Adachi M Kennett MJ Billin AN Willson TM PPARbeta/delta protects against experimental colitis through a ligand-independent mechanism. Dig Dis Sci (2007) 52:2912–9. 10.1007/s10620-006-9644-9 17404849

[150] Bassaganya-Riera J Hontecillas R CLA and n-3 PUFA differentially modulate clinical activity and colonic PPAR-responsive gene expression in a pig model of experimental IBD. Clin Nutr (2006) 25:454–65. 10.1016/j.clnu.2005.12.008 16698153

[151] Park BH Vogelstein B Kinzler KW Genetic disruption of PPARdelta decreases the tumorigenicity of human colon cancer cells. Proc Natl Acad Sci USA (2001) 98:2598–603. 10.1073/pnas.051630998 PMC30184 11226285

[152] Baaten BJ Li CR Deiro MF Lin MM Linton PJ Bradley LM CD44 regulates survival and memory development in Th1 cells. Immunity (2010) 32:104–15. 10.1016/j.immuni.2009.10.011 PMC2858628 20079666

[153] Harman FS Nicol CJ Marin HE Ward JM Gonzalez FJ Peters JM Peroxisome proliferator-activated receptor-delta attenuates colon carcinogenesis. Nat Med (2004) 10:481–3. 10.1038/nm1026 15048110

[154] Reed KR Sansom OJ Hayes AJ Gescher AJ Winton DJ Peters JM PPARdelta status and Apc-mediated tumourigenesis in the mouse intestine. Oncogene (2004) 23:8992–6. 10.1038/sj.onc.1208143 15480419

[155] Roy N Barnett M Knoch B Dommels Y McNabb W Nutrigenomics applied to an animal model of Inflammatory Bowel Diseases: transcriptomic analysis of the effects of eicosapentaenoic acid- and arachidonic acid-enriched diets. Mutat Res (2007) 622:103–16. 10.1016/j.mrfmmm.2007.04.003 17574631

[156] Langmann T Moehle C Mauerer R Scharl M Liebisch G Zahn A Loss of detoxification in inflammatory bowel disease: dysregulation of pregnane X receptor target genes. Gastroenterology (2004) 127:26–40. 10.1053/j.gastro.2004.04.019 15236169

[157] Lawrance IC Fiocchi C Chakravarti S Ulcerative colitis and Crohn’s disease: distinctive gene expression profiles and novel susceptibility candidate genes. Hum Mol Genet (2001) 10:445–56. 10.1093/hmg/10.5.445 11181568

[158] Dommels YE Butts CA Zhu S Davy M Martell S Hedderley D Characterization of intestinal inflammation and identification of related gene expression changes in mdr1a(-/-) mice. Genes Nutr (2007) 2:209–23. 10.1007/s12263-007-0051-4 PMC2474946 18850176

[159] Cao W Kayama H Chen ML Delmas A Sun A Kim SY The Xenobiotic Transporter Mdr1 Enforces T Cell Homeostasis in the Presence of Intestinal Bile Acids. Immunity (2017) 47:1182–96.e1110. 10.1016/j.immuni.2017.11.012 29262351 PMC5741099

[160] Hemmings BA Restuccia DF PI3K-PKB/Akt pathway. Cold Spring Harb Perspect Biol (2012) 4:a011189. 10.1101/cshperspect.a011189 22952397 PMC3428770

[161] Kim DH Kim JY Yu BP Chung HY The activation of NF-kappaB through Akt-induced FOXO1 phosphorylation during aging and its modulation by calorie restriction. Biogerontology (2008) 9:33–47. 10.1007/s10522-007-9114-6 17972158

[162] Panduro M Benoist C Mathis D Tissue Tregs. Annu Rev Immunol (2016) 34:609–33. 10.1146/annurev-immunol-032712-095948 PMC4942112 27168246

[163] Round JL Mazmanian SK Inducible Foxp3+ regulatory T-cell development by a commensal bacterium of the intestinal microbiota. Proc Natl Acad Sci USA (2010) 107:12204–9. 10.1073/pnas.0909122107 PMC2901479 20566854

[164] Arpaia N Campbell C Fan X Dikiy S van der Veeken J deRoos P Metabolites produced by commensal bacteria promote peripheral regulatory T-cell generation. Nature (2013) 504:451–5. 10.1038/nature12726 PMC3869884 24226773

[165] Sefik E Geva-Zatorsky N Oh S Konnikova L Zemmour D McGuire AM MUCOSAL IMMUNOLOGY. Individual intestinal symbionts induce a distinct population of RORgamma(+) regulatory T cells. Science (2015) 349:993–7. 10.1126/science.aaa9420 PMC4700932 26272906

[166] Zhou L Zhang M Wang Y Dorfman RG Liu H Yu T Faecalibacterium prausnitzii Produces Butyrate to Maintain Th17/Treg Balance and to Ameliorate Colorectal Colitis by Inhibiting Histone Deacetylase 1. Inflammation Bowel Dis (2018) 24:1926–40. 10.1093/ibd/izy182 29796620

[167] Hofmann AF Hagey LR Key discoveries in bile acid chemistry and biology and their clinical applications: history of the last eight decades. J Lipid Res (2014) 55:1553–95. 10.1194/jlr.R049437 PMC4109754 24838141

[168] Song X Sun X Oh SF Wu M Zhang Y Zheng W Microbial bile acid metabolites modulate gut RORgamma(+) regulatory T cell homeostasis. Nature (2020) 577:410–5. 10.1038/s41586-019-1865-0 PMC7274525 31875848

[169] Maciolek JA Pasternak JA Wilson HL Metabolism of activated T lymphocytes. Curr Opin Immunol (2014) 27:60–74. 10.1016/j.coi.2014.01.006 24556090

[170] Pompura SL Wagner A Kitz A Laperche J Yosef N Dominguez-Villar M Oleic acid restores suppressive defects in tissue-resident FOXP3 regulatory T cells from patients with multiple sclerosis. J Clin Invest (2021) 131(2):e138519. 10.1172/JCI138519 PMC7810477 33170805

[171] Pacella I Procaccini C Focaccetti C Miacci S Timperi E Faicchia D Fatty acid metabolism complements glycolysis in the selective regulatory T cell expansion during tumor growth. Proc Natl Acad Sci USA (2018) 115:E6546–55. 10.1073/pnas.1720113115 PMC6048537 29941600

[172] Rodriguez-Palacios A Bederman I Menghini P Omenetti S Pizarro T Cominelli F Tu1875 Medium-Chain Fatty Acid Lymphoid Organ Affinity and T Regulatory Cells in Experimental Crohn’s Disease. Gastroenterology (2016) 150:S965. 10.1016/S0016-5085(16)33269-3

[173] Bachem A Makhlouf C Binger KJ de Souza DP Tull D Hochheiser K Microbiota-Derived Short-Chain Fatty Acids Promote the Memory Potential of Antigen-Activated CD8(+) T Cells. Immunity 51:285–97.e285. 10.1016/j.immuni.2019.06.002(2019 31272808

[174] Cho JY Chi SG Chun HS Oral administration of docosahexaenoic acid attenuates colitis induced by dextran sulfate sodium in mice. Mol Nutr Food Res (2011) 55:239–46. 10.1002/mnfr.201000070 20824662

[175] Xiao L Sonne SB Feng Q Chen N Xia Z Li X High-fat feeding rather than obesity drives taxonomical and functional changes in the gut microbiota in mice. Microbiome (2017) 5:43. 10.1186/s40168-017-0258-6 28390422 PMC5385073

[176] Ley RE Turnbaugh PJ Klein S Gordon J I Microbial ecology: human gut microbes associated with obesity. Nature (2006) 444:1022–3. 10.1038/4441022a 17183309

[177] Clemente JC Ursell LK Parfrey LW Knight R The impact of the gut microbiota on human health: an integrative view. Cell (2012) 148:1258–70. 10.1016/j.cell.2012.01.035 PMC5050011 22424233

[178] Tremaroli V Backhed F Functional interactions between the gut microbiota and host metabolism. Nature (2012) 489:242–9. 10.1038/nature11552 22972297

[179] Zitomersky NL Atkinson BJ Franklin SW Mitchell PD Snapper SB Comstock LE Characterization of adherent bacteroidales from intestinal biopsies of children and young adults with inflammatory bowel disease. PloS One (2013) 8:e63686. 10.1371/journal.pone.0063686 23776434 PMC3679120

[180] Dziarski R Park SY Kashyap DR Dowd SE Gupta D Pglyrp-Regulated Gut Microflora Prevotella falsenii, Parabacteroides distasonis and Bacteroides eggerthii Enhance and Alistipes finegoldii Attenuates Colitis in Mice. PloS One (2016) 11:e0146162. 10.1371/journal.pone.0146162 26727498 PMC4699708

[181] Kverka M Zakostelska Z Klimesova K Sokol D Hudcovic T Hrncir T Oral administration of Parabacteroides distasonis antigens attenuates experimental murine colitis through modulation of immunity and microbiota composition. Clin Exp Immunol (2011) 163:250–9. 10.1111/j.1365-2249.2010.04286.x PMC3043316 21087444

[182] Mujico JR Baccan GC Gheorghe A Diaz LE Marcos A Changes in gut microbiota due to supplemented fatty acids in diet-induced obese mice. Br J Nutr (2013) 110:711–20. 10.1017/S0007114512005612 23302605

[183] Zheng J Wittouck S Salvetti E Franz C Harris HMB Mattarelli P A taxonomic note on the genus Lactobacillus: Description of 23 novel genera, emended description of the genus Lactobacillus Beijerinck 1901, and union of Lactobacillaceae and Leuconostocaceae. Int J Syst Evol Microbiol (2020) 70:2782–858. 10.1099/ijsem.0.004107 32293557

[184] Kankaanpaa PE Salminen SJ Isolauri E Lee YK The influence of polyunsaturated fatty acids on probiotic growth and adhesion. FEMS Microbiol Lett (2001) 194:149–53. 10.1111/j.1574-6968.2001.tb09460.x 11164299

[185] Ohland CL Kish L Bell H Thiesen A Hotte N Pankiv E Effects of Lactobacillus helveticus on murine behavior are dependent on diet and genotype and correlate with alterations in the gut microbiome. Psychoneuroendocrinology (2013) 38:1738–47. 10.1016/j.psyneuen.2013.02.008 23566632

[186] Mencarelli A Distrutti E Renga B D’Amore C Cipriani S Palladino G Probiotics modulate intestinal expression of nuclear receptor and provide counter-regulatory signals to inflammation-driven adipose tissue activation. PloS One (2011) 6:e22978. 10.1371/journal.pone.0022978 21829567 PMC3146529

[187] McMahon MD Prather KL Functional screening and in vitro analysis reveal thioesterases with enhanced substrate specificity profiles that improve short-chain fatty acid production in Escherichia coli. Appl Environ Microbiol (2014) 80:1042–50. 10.1128/AEM.03303-13 PMC3911193 24271180

[188] Zhao C Dong H Zhang Y Li Y Discovery of potential genes contributing to the biosynthesis of short-chain fatty acids and lactate in gut microbiota from systematic investigation in E. coli. NPJ Biofilms Microbiomes (2019) 5:19. 10.1038/s41522-019-0092-7 31312512 PMC6626047

[189] Ewaschuk JB Walker JW Diaz H Madsen KL Bioproduction of conjugated linoleic acid by probiotic bacteria occurs in vitro and in vivo in mice. J Nutr (2006) 136:1483–7. 10.1093/jn/136.6.1483 16702308

[190] Parada Venegas D De la Fuente MK Landskron G Gonzalez MJ Quera R Dijkstra G Short Chain Fatty Acids (SCFAs)-Mediated Gut Epithelial and Immune Regulation and Its Relevance for Inflammatory Bowel Diseases. Front Immunol (2019) 10:277. 10.3389/fimmu.2019.00277 30915065 PMC6421268

[191] Zhao L Huang Y Lu L Yang W Huang T Lin Z Saturated long-chain fatty acid-producing bacteria contribute to enhanced colonic motility in rats. Microbiome (2018) 6:107. 10.1186/s40168-018-0492-6 29903041 PMC6003035

[192] Parker B Wearsch P Veloo A Rodriguez-Palacios A The genus Alistipes: Gut bacteria with emerging implications to inflammation, cancer and mental health. Front Immunol (2020) 11(906):1–15. 10.3389/fimmu.2020.00906 32582143 PMC7296073

[193] Portela DS Vieira TO Matos SM de Oliveira NF Vieira GO Maternal obesity, environmental factors, cesarean delivery and breastfeeding as determinants of overweight and obesity in children: results from a cohort. BMC Pregnancy Childbirth (2015) 15:94. 10.1186/s12884-015-0518-z 25884808 PMC4407299

[194] Bibi S Kang Y Du M Zhu MJ Maternal high-fat diet consumption enhances offspring susceptibility to DSS-induced colitis in mice. Obes (Silver Spring) (2017) 25:901–8. 10.1002/oby.21816 PMC6461699 28339172

[195] Xie R Sun Y Wu J Huang S Jin G Guo Z Maternal High Fat Diet Alters Gut Microbiota of Offspring and Exacerbates DSS-Induced Colitis in Adulthood. Front Immunol (2018) 9:2608. 10.3389/fimmu.2018.02608 30483266 PMC6243010

[196] Babu ST Niu X Raetz M Savani RC Hooper LV Mirpuri J Maternal high-fat diet results in microbiota-dependent expansion of ILC3s in mice offspring. JCI Insight (2018) 3(19):e99223. 10.1172/jci.insight.99223 PMC6237468 30282818

[197] Srinivasan M Katewa SD Palaniyappan A Pandya JD Patel MS Maternal high-fat diet consumption results in fetal malprogramming predisposing to the onset of metabolic syndrome-like phenotype in adulthood. Am J Physiol Endocrinol Metab (2006) 291:E792–99. 10.1152/ajpendo.00078.2006 16720630

[198] Jacobson K Mundra H Innis SM Intestinal responsiveness to experimental colitis in young rats is altered by maternal diet Am J Physiol Gastrointest Liver Physiol (2005) 289:G13–20. 10.1152/ajpgi.00292.2001 15731507

[199] Ohtsuka Y Okada K Yamakawa Y Ikuse T Baba Y Inage E omega-3 fatty acids attenuate mucosal inflammation in premature rat pups. J Pediatr Surg (2011) 46:489–95. 10.1016/j.jpedsurg.2010.07.032 21376198

[200] Lu J Jilling T Li D Caplan MS Polyunsaturated fatty acid supplementation alters proinflammatory gene expression and reduces the incidence of necrotizing enterocolitis in a neonatal rat model. Pediatr Res (2007) 61:427–32. 10.1203/pdr.0b013e3180332ca5 PMC2675177 17515866

[201] Chawla A Karl P II Fisher SE Effect of N-3 polyunsaturated fatty acid supplemented diet on neutrophil-mediated ileal permeability and neutrophil function in the rat. J Am Coll Nutr (1995) 14:258–63. 10.1080/07315724.1995.10718505 8586775

[202] Camuesco D Galvez J Nieto A Comalada M Rodriguez-Cabezas ME Concha A Dietary olive oil supplemented with fish oil, rich in EPA and DHA (n-3) polyunsaturated fatty acids, attenuates colonic inflammation in rats with DSS-induced colitis. J Nutr (2005) 135:687–94. 10.1093/jn/135.4.687 15795419

[203] Gill R Tsung A Billiar T Linking oxidative stress to inflammation: Toll-like receptors. Free Radic Biol Med (2010) 48:1121–32. 10.1016/j.freeradbiomed.2010.01.006 PMC3423196 20083193

[204] Mah R Thomas JR Shafer CM Drug discovery considerations in the development of covalent inhibitors. Bioorg Med Chem Lett (2014) 24:33–9. 10.1016/j.bmcl.2013.10.003 24314671

[205] Menon D Coll R O’Neill LA Board PG GSTO1-1 modulates metabolism in macrophages activated through the LPS and TLR4 pathway. J Cell Sci (2015) 128:1982–90. 10.1242/jcs.167858 25908843

[206] Everard A Geurts L Caesar R Van Hul M Matamoros S Duparc T Intestinal epithelial MyD88 is a sensor switching host metabolism towards obesity according to nutritional status. Nat Commun (2014) 5:5648. 10.1038/ncomms6648 25476696 PMC4268705

[207] Jia L Vianna CR Fukuda M Berglund ED Liu C Tao C Hepatocyte Toll-like receptor 4 regulates obesity-induced inflammation and insulin resistance. Nat Commun (2014) 5:3878. 10.1038/ncomms4878 24815961 PMC4080408

[208] Menon D Coll R O’Neill LA Board PG Glutathione transferase omega 1 is required for the lipopolysaccharide-stimulated induction of NADPH oxidase 1 and the production of reactive oxygen species in macrophages. Free Radic Biol Med (2014) 73:318–27. 10.1016/j.freeradbiomed.2014.05.020 24873723

[209] Tannahill GM Curtis AM Adamik J Palsson-McDermott EM McGettrick AF Goel G Succinate is an inflammatory signal that induces IL-1beta through HIF-1alpha. Nature (2013) 496:238–42. 10.1038/nature11986 PMC4031686 23535595

[210] Menon D Innes A Oakley AJ Dahlstrom JE Jensen LM Brustle A GSTO1-1 plays a pro-inflammatory role in models of inflammation, colitis and obesity. Sci Rep (2017) 7:17832. 10.1038/s41598-017-17861-6 29259211 PMC5736720

[211] Jostins L Ripke S Weersma RK Duerr RH McGovern DP Hui KY Host-microbe interactions have shaped the genetic architecture of inflammatory bowel disease. Nature (2012) 491:119–24. 10.1038/nature11582 PMC3491803 23128233

[212] Kagan VE Mao G Qu F Angeli JP Doll S Croix CS Oxidized arachidonic and adrenic PEs navigate cells to ferroptosis. Nat Chem Biol (2017) 13:81–90. 10.1038/nchembio.2238 27842066 PMC5506843

[213] Doll S Proneth B Tyurina YY Panzilius E Kobayashi S Ingold I ACSL4 dictates ferroptosis sensitivity by shaping cellular lipid composition. Nat Chem Biol (2017) 13:91–8. 10.1038/nchembio.2239 PMC5610546 27842070

[214] Mayr L Grabherr F Schwarzler J Reitmeier I Sommer F Gehmacher T Dietary lipids fuel GPX4-restricted enteritis resembling Crohn’s disease. Nat Commun (2020) 11:1775. 10.1038/s41467-020-15646-6 32286299 PMC7156516

[215] Kamerkar S LeBleu VS Sugimoto H Yang S Ruivo CF Melo SA Exosomes facilitate therapeutic targeting of oncogenic KRAS in pancreatic cancer. Nature (2017) 546:498–503. 10.1038/nature22341 28607485 PMC5538883

[216] Colombo M Raposo G Thery C Biogenesis, secretion, and intercellular interactions of exosomes and other extracellular vesicles. Annu Rev Cell Dev Biol (2014) 30:255–89. 10.1146/annurev-cellbio-101512-122326 25288114

[217] Hoshino A Costa-Silva B Shen TL Rodrigues G Hashimoto A Tesic Mark M Tumour exosome integrins determine organotropic metastasis. Nature (2015) 527:329–35. 10.1038/nature15756 PMC4788391 26524530

[218] Thomou T Mori MA Dreyfuss JM Konishi M Sakaguchi M Wolfrum C Adipose-derived circulating miRNAs regulate gene expression in other tissues. Nature (2017) 542:450–5. 10.1038/nature21365 PMC5330251 28199304

[219] Isola AL Chen S Exosomes: The Messengers of Health and Disease. Curr Neuropharmacol (2017) 15:157–65. 10.2174/1570159x14666160825160421 PMC5327461 27568544

[220] Wei M Gao X Liu L Li Z Wan Z Dong Y Visceral Adipose Tissue Derived Exosomes Exacerbate Colitis Severity via Pro-inflammatory MiRNAs in High Fat Diet Fed Mice. ACS Nano (2020) 14:5099–110. 10.1021/acsnano.0c01860 32275391

[221] Brandl K Rutschmann S Li X Du X Xiao N Schnabl B Enhanced sensitivity to DSS colitis caused by a hypomorphic Mbtps1 mutation disrupting the ATF6-driven unfolded protein response. Proc Natl Acad Sci USA (2009) 106:3300–5. 10.1073/pnas.0813036106 PMC2651297 19202076

[222] Kaser A Lee AH Franke A Glickman JN Zeissig S Tilg H XBP1 links ER stress to intestinal inflammation and confers genetic risk for human inflammatory bowel disease. Cell (2008) 134:743–56. 10.1016/j.cell.2008.07.021 PMC2586148 18775308

[223] McGuckin MA Linden SK Sutton P Florin TH Mucin dynamics and enteric pathogens. Nat Rev Microbiol (2011) 9:265–78. 10.1038/nrmicro2538 21407243

[224] Walter P Ron D The unfolded protein response: from stress pathway to homeostatic regulation. Science (2011) 334:1081–6. 10.1126/science.1209038 22116877

[225] Wei X Yang Z Rey FE Ridaura VK Davidson NO Gordon J I Fatty acid synthase modulates intestinal barrier function through palmitoylation of mucin 2. Cell Host Microbe (2012) 11:140–52. 10.1016/j.chom.2011.12.006 PMC3285413 22341463

[226] Hasnain SZ Borg DJ Harcourt BE Tong H Sheng YH Ng CP Glycemic control in diabetes is restored by therapeutic manipulation of cytokines that regulate beta cell stress. Nat Med (2014) 20:1417–26. 10.1038/nm.3705 25362253

[227] Hasnain SZ Tauro S Das I Tong H Chen AC Jeffery PL IL-10 promotes production of intestinal mucus by suppressing protein misfolding and endoplasmic reticulum stress in goblet cells. Gastroenterology (2013) 144:357–368 e359. 10.1053/j.gastro.2012.10.043 23123183

[228] Rothhammer V Quintana FJ The aryl hydrocarbon receptor: an environmental sensor integrating immune responses in health and disease. Nat Rev Immunol (2019) 19:184–97. 10.1038/s41577-019-0125-8 30718831

[229] Bernshausen T Jux B Esser C Abel J Fritsche E Tissue distribution and function of the Aryl hydrocarbon receptor repressor (AhRR) in C57BL/6 and Aryl hydrocarbon receptor deficient mice. Arch Toxicol (2006) 80:206–11. 10.1007/s00204-005-0025-5 16205913

[230] Li Y Innocentin S Withers DR Roberts NA Gallagher AR Grigorieva EF Exogenous stimuli maintain intraepithelial lymphocytes via aryl hydrocarbon receptor activation. Cell (2011) 147:629–40. 10.1016/j.cell.2011.09.025 21999944

[231] Schanz O Chijiiwa R Cengiz SC Majlesain Y Weighardt H Takeyama H Dietary AhR Ligands Regulate AhRR Expression in Intestinal Immune Cells and Intestinal Microbiota Composition. Int J Mol Sci (2020) 21(9):3189. 10.3390/ijms21093189 PMC7246938 32366032

[232] Biagioli M Carino A Fiorucci C Annunziato G Marchiano S Bordoni M The Aryl Hydrocarbon Receptor (AhR) Mediates the Counter-Regulatory Effects of Pelargonidins in Models of Inflammation and Metabolic Dysfunctions. Nutrients (2019) 11(8):1820. 10.3390/nu11081820 PMC6723439 31394746

[233] Ghattamaneni NK Sharma A Panchal SK Brown L Pelargonidin 3-glucoside-enriched strawberry attenuates symptoms of DSS-induced inflammatory bowel disease and diet-induced metabolic syndrome in rats. Eur J Nutr (2020) 59:2905–18. 10.1007/s00394-019-02130-1 31696323

[234] Davey MW Stals E Panis B Keulemans J Swennen RL High-throughput determination of malondialdehyde in plant tissues. Anal Biochem (2005) 347:201–7. 10.1016/j.ab.2005.09.041 16289006

[235] Pryor WA Stanley JP Letter: A suggested mechanism for the production of malonaldehyde during the autoxidation of polyunsaturated fatty acids. Nonenzymatic production of prostaglandin endoperoxides during autoxidation. J Org Chem (1975) 40:3615–7. 10.1021/jo00912a038 1185332

[236] Kanner J Dietary advanced lipid oxidation endproducts are risk factors to human health. Mol Nutr Food Res (2007) 51:1094–101. 10.1002/mnfr.200600303 17854006

[237] Martinez-Useros J Garcia-Foncillas J Obesity and colorectal cancer: molecular features of adipose tissue. J Transl Med (2016) 14:21. 10.1186/s12967-016-0772-5 26801617 PMC4722674

[238] Tuzun A Erdil A Inal V Aydin A Bagci S Yesilova Z Oxidative stress and antioxidant capacity in patients with inflammatory bowel disease. Clin Biochem (2002) 35:569–72. 10.1016/s0009-9120(02)00361-2 12493587

[239] Alzoghaibi MA Walsh SW Willey A Fowler AA,3 Graham MF Linoleic acid, but not oleic acid, upregulates the production of interleukin-8 by human intestinal smooth muscle cells isolated from patients with Crohn’s disease. Clin Nutr (2003) 22:529–35. 10.1016/s0261-5614(03)00083-9 14613754

[240] Alzoghaibi MA Al Mofleh IA Al-Jebreen AM Lipid peroxides in patients with inflammatory bowel disease. Saudi J Gastroenterol (2007) 13:187–90. 10.4103/1319-3767.36750 19858644

[241] Yoo JS Park CY Seo YK Woo SH Kim DY Han SN Vitamin D supplementation partially affects colonic changes in dextran sulfate sodium-induced colitis obese mice but not lean mice. Nutr Res (2019) 67:90–9. 10.1016/j.nutres.2019.03.009 30995974

[242] Suzuki T Hara H Dietary fat and bile juice, but not obesity, are responsible for the increase in small intestinal permeability induced through the suppression of tight junction protein expression in LETO and OLETF rats. Nutr Metab (Lond) (2010) 7:19. 10.1186/1743-7075-7-19 20222989 PMC2848226

[243] Stenman LK Holma R Gylling H Korpela R Genetically obese mice do not show increased gut permeability or faecal bile acid hydrophobicity. Br J Nutr (2013) 110:1157–64. 10.1017/S000711451300024X 23442231

[244] Tanaka S Nemoto Y Takei Y Morikawa R Oshima S Nagaishi T High-fat diet-derived free fatty acids impair the intestinal immune system and increase sensitivity to intestinal epithelial damage. Biochem Biophys Res Commun (2020) 522:971–7. 10.1016/j.bbrc.2019.11.158 31810607

[245] Moore TA Moore BB Newstead MW Standiford TJ Gamma delta-T cells are critical for survival and early proinflammatory cytokine gene expression during murine Klebsiella pneumonia. J Immunol (2000) 165:2643–50. 10.4049/jimmunol.165.5.2643 10946293

[246] Mombaerts P Mizoguchi E Grusby MJ Glimcher LH Bhan AK Tonegawa S Spontaneous development of inflammatory bowel disease in T cell receptor mutant mice. Cell (1993) 75:274–82. 10.1016/0092-8674(93)80069-q 8104709

[247] Taupin D Podolsky DK Trefoil factors: initiators of mucosal healing. Nat Rev Mol Cell Biol (2003) 4:721–32. 10.1038/nrm1203 14506475

[248] Raimondi F Santoro P Barone MV Pappacoda S Barretta ML Nanayakkara M Bile acids modulate tight junction structure and barrier function of Caco-2 monolayers via EGFR activation. Am J Physiol Gastrointest Liver Physiol (2008) 294:G906–13. 10.1152/ajpgi.00043.2007 18239063

[249] Stenman LK Holma R Eggert A Korpela R A novel mechanism for gut barrier dysfunction by dietary fat: epithelial disruption by hydrophobic bile acids. Am J Physiol Gastrointest Liver Physiol (2013) 304:G227–34. 10.1152/ajpgi.00267.2012 23203158

[250] Bernstein H Holubec H Bernstein C Ignatenko NA Gerner E Dvorak K Deoxycholate-induced colitis is markedly attenuated in Nos2 knockout mice in association with modulation of gene expression profiles. Dig Dis Sci (2007) 52:628–42. 10.1007/s10620-006-9608-0 17253130

[251] Zhao S Gong Z Zhou J Tian C Gao Y Xu C Deoxycholic Acid Triggers NLRP3 Inflammasome Activation and Aggravates DSS-Induced Colitis in Mice. Front Immunol (2016) 7:536. 10.3389/fimmu.2016.00536 27965665 PMC5124666

[252] Liu JZ van Sommeren S Huang H Ng SC Alberts R Takahashi A Association analyses identify 38 susceptibility loci for inflammatory bowel disease and highlight shared genetic risk across populations. Nat Genet (2015) 47:979–86. 10.1038/ng.3359 PMC4881818 26192919

[253] Cox DG Crusius JB Peeters PH Bueno-de-Mesquita HB Pena AS Canzian F Haplotype of prostaglandin synthase 2/cyclooxygenase 2 is involved in the susceptibility to inflammatory bowel disease. World J Gastroenterol (2005) 11:6003–8. 10.3748/wjg.v11.i38.6003 PMC4436724 16273614

[254] de Vries HS te Morsche RH van Oijen MG Nagtegaal ID Peters WH de Jong DJ The functional -765G–>C polymorphism of the COX-2 gene may reduce the risk of developing crohn’s disease. PloS One (2010) 5:e15011. 10.1371/journal.pone.0015011 21124790 PMC2991351

[255] Newberry RD Stenson WF Lorenz RG Cyclooxygenase-2-dependent arachidonic acid metabolites are essential modulators of the intestinal immune response to dietary antigen. Nat Med (1999) 5:900–6. 10.1038/11341 10426313

[256] Newberry RD McDonough JS Stenson WF Lorenz RG Spontaneous and continuous cyclooxygenase-2-dependent prostaglandin E2 production by stromal cells in the murine small intestine lamina propria: directing the tone of the intestinal immune response. J Immunol (2001) 166:4465–72. 10.4049/jimmunol.166.7.4465 11254702

[257] Lin JA Watanabe J Rozengurt N Narasimha A Martin MG Wang J Atherogenic diet causes lethal ileo-ceco-colitis in cyclooxygenase-2 deficient mice. Prostaglandins Other Lipid Mediat (2007) 84:98–107. 10.1016/j.prostaglandins.2007.04.004 17991612 PMC2701900

[258] Watanabe J Lin JA Narasimha AJ Shahbazian A Ishikawa TO Martin MG Novel anti-inflammatory functions for endothelial and myeloid cyclooxygenase-2 in a new mouse model of Crohn’s disease. Am J Physiol Gastrointest Liver Physiol (2010) 298:G842–50. 10.1152/ajpgi.00468.2009 PMC8875131 20299600

[259] Fukunaga K Kohli P Bonnans C Fredenburgh LE Levy BD Cyclooxygenase 2 plays a pivotal role in the resolution of acute lung injury. J Immunol (2005) 174:5033–9. 10.4049/jimmunol.174.8.5033 15814734

[260] Norris PC Gosselin D Reichart D Glass CK Dennis EA Phospholipase A2 regulates eicosanoid class switching during inflammasome activation. Proc Natl Acad Sci USA (2014) 111:12746–51. 10.1073/pnas.1404372111 PMC4156727 25139986

[261] Gravaghi C La Perle KM Ogrodwski P Kang JX Quimby F Lipkin M Cox-2 expression, PGE(2) and cytokines production are inhibited by endogenously synthesized n-3 PUFAs in inflamed colon of fat-1 mice. J Nutr Biochem (2011) 22:360–5. 10.1016/j.jnutbio.2010.03.003 20655721

[262] Lee JY Plakidas A Lee WH Heikkinen A Chanmugam P Bray G Differential modulation of Toll-like receptors by fatty acids: preferential inhibition by n-3 polyunsaturated fatty acids. J Lipid Res (2003) 44:479–86. 10.1194/jlr.M200361-JLR200 12562875

[263] Dommels YE Haring MM Keestra NG Alink GM van Bladeren PJ van Ommen B The role of cyclooxygenase in n-6 and n-3 polyunsaturated fatty acid mediated effects on cell proliferation, PGE(2) synthesis and cytotoxicity in human colorectal carcinoma cell lines. Carcinogenesis (2003) 24:385–92. 10.1093/carcin/24.3.385 12663496

[264] Stulnig TM Immunomodulation by polyunsaturated fatty acids: mechanisms and effects. Int Arch Allergy Immunol (2003) 132:310–21. 10.1159/000074898 14707462

[265] Guarner F Vilaseca J Malagelada JR Dietary manipulation in experimental inflammatory bowel disease. Agents Actions (1992) 236:C10–14. Spec No. 10.1007/BF01996088 1442325

[266] Mascolo N Izzo AA Autore G Maiello FM Di Carlo G Capasso F Acetic acid-induced colitis in normal and essential fatty acid deficient rats. J Pharmacol Exp Ther (1995) 272:469–75. 7815363

[267] Gil A Polyunsaturated fatty acids and inflammatory diseases. BioMed Pharmacother (2002) 56:388–96. 10.1016/s0753-3322(02)00256-1 12442911

[268] Wallace JL Eicosanoids in the gastrointestinal tract. Br J Pharmacol (2019) 176:1000–8. 10.1111/bph.14178 PMC6451073 29485681

[269] Li C Wu X Liu S Shen D Zhu J Liu K Role of Resolvins in the Inflammatory Resolution of Neurological Diseases. Front Pharmacol (2020) 11:612. 10.3389/fphar.2020.00612 32457616 PMC7225325

[270] Schwanke RC Marcon R Bento AF Calixto JB EPA- and DHA-derived resolvins’ actions in inflammatory bowel disease. Eur J Pharmacol (2016) 785:156–64. 10.1016/j.ejphar.2015.08.050 26325092

[271] Bannenberg GL Chiang N Ariel A Arita M Tjonahen E Gotlinger KH Molecular circuits of resolution: formation and actions of resolvins and protectins. J Immunol (2005) 174:4345–55. 10.4049/jimmunol.174.7.4345 15778399

[272] Ariel A Li PL Wang W Tang WX Fredman G Hong S The docosatriene protectin D1 is produced by TH2 skewing and promotes human T cell apoptosis via lipid raft clustering. J Biol Chem (2005) 280:43079–86. 10.1074/jbc.M509796200 16216871

[273] Arita M Bianchini F Aliberti J Sher A Chiang N Hong S Stereochemical assignment, antiinflammatory properties, and receptor for the omega-3 lipid mediator resolvin E1. J Exp Med (2005) 201:713–22. 10.1084/jem.20042031 PMC2212834 15753205

[274] Campbell EL MacManus CF Kominsky DJ Keely S Glover LE Bowers BE Resolvin E1-induced intestinal alkaline phosphatase promotes resolution of inflammation through LPS detoxification. Proc Natl Acad Sci USA (2010) 107:14298–303. 10.1073/pnas.0914730107 PMC2922533 20660763

[275] Kohnke T Gomolka B Bilal S Zhou X Sun Y Rothe M Acetylsalicylic Acid reduces the severity of dextran sodium sulfate-induced colitis and increases the formation of anti-inflammatory lipid mediators. BioMed Res Int (2013) 2013:748160. 10.1155/2013/748160 24083240 PMC3780524

[276] Serhan CN Levy BD Resolvins in inflammation: emergence of the pro-resolving superfamily of mediators. J Clin Invest (2018) 128:2657–69. 10.1172/JCI97943 PMC6025982 29757195

[277] Kamitani H Ikawa H Hsi LC Watanabe T DuBois RN Eling TE Regulation of 12-lipoxygenase in rat intestinal epithelial cells during differentiation and apoptosis induced by sodium butyrate. Arch Biochem Biophys (1999) 368:45–55. 10.1006/abbi.1999.1284 10415110

[278] Meriwether D Sulaiman D Volpe C Dorfman A Grijalva V Dorreh N Apolipoprotein A-I mimetics mitigate intestinal inflammation in COX2-dependent inflammatory bowel disease model. J Clin Invest (2019) 129:3670–85. 10.1172/JCI123700 PMC6715371 31184596

[279] Ahima RS Flier JS Adipose tissue as an endocrine organ. Trends Endocrinol Metab (2000) 11:327–32. 10.1016/s1043-2760(00)00301-5 10996528

[280] Bouloumie A Curat CA Sengenes C Lolmede K Miranville A Busse R Role of macrophage tissue infiltration in metabolic diseases. Curr Opin Clin Nutr Metab Care (2005) 8:347–54. 10.1097/01.mco.0000172571.41149.52 15930956

[281] Bruun JM Verdich C Toubro S Astrup A Richelsen B Association between measures of insulin sensitivity and circulating levels of interleukin-8, interleukin-6 and tumor necrosis factor-alpha. Effect of weight loss in obese men. Eur J Endocrinol (2003) 148:535–42. 10.1530/eje.0.1480535 12720537

[282] Kern PA Saghizadeh M Ong JM Bosch RJ Deem R Simsolo RB The expression of tumor necrosis factor in human adipose tissue. Regulation by obesity, weight loss, and relationship to lipoprotein lipase. J Clin Invest (1995) 95:2111–9. 10.1172/JCI117899 PMC295809 7738178

[283] Skurk T Alberti-Huber C Herder C Hauner H Relationship between adipocyte size and adipokine expression and secretion. J Clin Endocrinol Metab (2007) 92:1023–33. 10.1210/jc.2006-1055 17164304

[284] Weisberg SP McCann D Desai M Rosenbaum M Leibel RL Ferrante AW Jr Obesity is associated with macrophage accumulation in adipose tissue. J Clin Invest (2003) 112:1796–808. 10.1172/JCI19246 PMC296995 14679176

[285] Neumeier M Weigert J Schaffler A Wehrwein G Muller-Ladner U Scholmerich J Different effects of adiponectin isoforms in human monocytic cells. J Leukoc Biol (2006) 79:803–8. 10.1189/jlb.0905521 16434692

[286] Sennello JA Fayad R Pini M Gove ME Fantuzzi G Transplantation of wild-type white adipose tissue normalizes metabolic, immune and inflammatory alterations in leptin-deficient ob/ob mice. Cytokine (2006) 36:261–6. 10.1016/j.cyto.2007.02.001 PMC1968154 17368040

[287] Cao H Adipocytokines in obesity and metabolic disease. J Endocrinol (2014) 220:T47–59. 10.1530/JOE-13-0339 PMC3887367 24403378

[288] Desreumaux P Ernst O Geboes K Gambiez L Berrebi D Muller-Alouf H Inflammatory alterations in mesenteric adipose tissue in Crohn’s disease. Gastroenterology (1999) 117:73–81. 10.1016/s0016-5085(99)70552-4 10381912

[289] Buning C von Kraft C Hermsdorf M Gentz E Wirth EK Valentini L Visceral Adipose Tissue in Patients with Crohn’s Disease Correlates with Disease Activity, Inflammatory Markers, and Outcome. Inflammation Bowel Dis (2015) 21:2590–7. 10.1097/MIB.0000000000000527 26222339

[290] Zulian A Cancello R Micheletto G Gentilini D Gilardini L Danelli P Visceral adipocytes: old actors in obesity and new protagonists in Crohn’s disease? Gut (2012) 61:86–94. 10.1136/gutjnl-2011-300391 21930728

[291] Weidinger C Ziegler JF Letizia M Schmidt F Siegmund B Adipokines and Their Role in Intestinal Inflammation. Front Immunol (2018) 9:1974. 10.3389/fimmu.2018.01974 30369924 PMC6194904

[292] Masaki T Chiba S Tatsukawa H Yasuda T Noguchi H Seike M Adiponectin protects LPS-induced liver injury through modulation of TNF-alpha in KK-Ay obese mice. Hepatology (2004) 40:177–84. 10.1002/hep.20282 15239101

[293] Nishihara T Matsuda M Araki H Oshima K Kihara S Funahashi T Effect of adiponectin on murine colitis induced by dextran sulfate sodium. Gastroenterology (2006) 131:853–61. 10.1053/j.gastro.2006.06.015 16952554

[294] Yamamoto K Kiyohara T Murayama Y Kihara S Okamoto Y Funahashi T Production of adiponectin, an anti-inflammatory protein, in mesenteric adipose tissue in Crohn’s disease. Gut (2005) 54:789–96. 10.1136/gut.2004.046516 PMC1774527 15888786

[295] Fayad R Pini M Sennello JA Cabay RJ Chan L Xu A Adiponectin deficiency protects mice from chemically induced colonic inflammation. Gastroenterology (2007) 132:601–14. 10.1053/j.gastro.2006.11.026 17258715

[296] Ogunwobi OO Beales IL Adiponectin stimulates proliferation and cytokine secretion in colonic epithelial cells. Regul Pept (2006) 134:105–13. 10.1016/j.regpep.2006.02.001 16529829

[297] McCaskey SJ Rondini EA Langohr IM Fenton J II Differential effects of energy balance on experimentally-induced colitis. World J Gastroenterol (2012) 18:627–36. 10.3748/wjg.v18.i7.627 PMC3281219 22363133

[298] Ballinger A Kelly P Hallyburton E Besser R Farthing M Plasma leptin in chronic inflammatory bowel disease and HIV: implications for the pathogenesis of anorexia and weight loss. Clin Sci (Lond) (1998) 94:479–83. 10.1042/cs0940479 9682669

[299] Franchimont D Roland S Gustot T Quertinmont E Toubouti Y Gervy MC Impact of infliximab on serum leptin levels in patients with Crohn’s disease. J Clin Endocrinol Metab (2005) 90:3510–6. 10.1210/jc.2004-1222 15784704

[300] Singh UP Singh NP Guan H Busbee B Price RL Taub DD The emerging role of leptin antagonist as potential therapeutic option for inflammatory bowel disease. Int Rev Immunol (2014) 33:23–33. 10.3109/08830185.2013.809071 23841494 PMC4159716

[301] Siegmund B Sennello JA Lehr HA Batra A Fedke I Zeitz M Development of intestinal inflammation in double IL-10- and leptin-deficient mice. J Leukoc Biol (2004) 76:782–6. 10.1189/jlb.0404239 15240754

[302] Zumbach MS Boehme MW Wahl P Stremmel W Ziegler R Nawroth PP Tumor necrosis factor increases serum leptin levels in humans. J Clin Endocrinol Metab (1997) 82:4080–2. 10.1210/jcem.82.12.4408 9398717

[303] Finck BN Kelley KW Dantzer R Johnson RW In vivo and in vitro evidence for the involvement of tumor necrosis factor-alpha in the induction of leptin by lipopolysaccharide. Endocrinology (1998) 139:2278–83. 10.1210/endo.139.5.6012 9564834

[304] Rosaspina S Liguori G Anzanel D Finzi G Salvatorelli G [Experimental tests of a microwave sterilization system]. Minerva Stomatol (1994) 43:17–21. 8170448

[305] Tiaka EK Manolakis AC Kapsoritakis AN Potamianos SP Unraveling the link between leptin, ghrelin and different types of colitis. Ann Gastroenterol (2011) 24:20–8. PMC3959465 24714276

[306] Chen YT Tsai SH Sheu SY Tsai LH Ghrelin improves LPS-induced gastrointestinal motility disturbances: roles of NO and prostaglandin E2. Shock (2010) 33:205–12. 10.1097/SHK.0b013e3181ae841b 19503023

[307] Waseem T Duxbury M Ito H Ashley SW Robinson MK Exogenous ghrelin modulates release of pro-inflammatory and anti-inflammatory cytokines in LPS-stimulated macrophages through distinct signaling pathways. Surgery (2008) 143:334–42. 10.1016/j.surg.2007.09.039 PMC2278045 18291254

[308] Zhao D Zhan Y Zeng H Moyer MP Mantzoros CS Pothoulakis C Ghrelin stimulates interleukin-8 gene expression through protein kinase C-mediated NF-kappaB pathway in human colonic epithelial cells. J Cell Biochem (2006) 97:1317–27. 10.1002/jcb.20744 16552751

[309] Gonzalez-Rey E Chorny A Delgado M Therapeutic action of ghrelin in a mouse model of colitis. Gastroenterology (2006) 130:1707–20. 10.1053/j.gastro.2006.01.041 16697735

[310] Konturek PC Brzozowski T Engel M Burnat G Gaca P Kwiecien S Ghrelin ameliorates colonic inflammation. Role of nitric oxide and sensory nerves. J Physiol Pharmacol (2009) 60:41–7. 10.1007/s12272-012-0714-6 19617644

[311] De Smet B Thijs T Moechars D Colsoul B Polders L Ver Donck L Endogenous and exogenous ghrelin enhance the colonic and gastric manifestations of dextran sodium sulphate-induced colitis in mice. Neurogastroenterol Motil (2009) 21:59–70. 10.1111/j.1365-2982.2008.01184.x 18823291

[312] Symonds EL Riedel CU O’Mahony D Lapthorne S O’Mahony L Shanahan F Involvement of T helper type 17 and regulatory T cell activity in Citrobacter rodentium invasion and inflammatory damage. Clin Exp Immunol (2009) 157:148–54. 10.1111/j.1365-2249.2009.03934.x PMC2710602 19659780

[313] Karagiannides I Kokkotou E Tansky M Tchkonia T Giorgadze N O’Brien M Induction of colitis causes inflammatory responses in fat depots: evidence for substance P pathways in human mesenteric preadipocytes. Proc Natl Acad Sci USA (2006) 103:5207–12. 10.1073/pnas.0600821103 PMC1458819 16549770

[314] Karagiannides I Torres D Tseng YH Bowe C Carvalho E Espinoza D Substance P as a novel anti-obesity target. Gastroenterology (2008) 134:747–55. 10.1053/j.gastro.2007.12.032 PMC2359157 18325388

[315] Basson A LaSalla A Lam G Kulpins D Moen E Sundrud M Artificial microbiome heterogeneity spurs six practical action themes and examples to increase study power-driven reproducibility. Sci Rep (2019) 10. 10.1038/s41598-020-60900-y Article number: 5039. PMC7081340 32193395

